# A Computational Profiling of Changes in Gene Expression and Transcription Factors Induced by vFLIP K13 in Primary Effusion Lymphoma

**DOI:** 10.1371/journal.pone.0037498

**Published:** 2012-05-18

**Authors:** Vasu Punj, Hittu Matta, Preet M. Chaudhary

**Affiliations:** 1 From Jane Anne Nohl Division of Hematology and Center for the Study of Blood Diseases, University of Southern California Keck School of Medicine, Los Angeles, California, United States of America; 2 Bioinformatics Core, Norris Comprehensive Cancer Center at USC Epigenome Center, University of Southern California Keck School of Medicine, Los Angeles, California, United States of America; Yale Medical School, United States of America

## Abstract

Infection with Kaposi's sarcoma associated herpesvirus (KSHV) has been linked to the development of primary effusion lymphoma (PEL), a rare lymphoproliferative disorder that is characterized by loss of expression of most B cell markers and effusions in the body cavities. This unique clinical presentation of PEL has been attributed to their distinctive plasmablastic gene expression profile that shows overexpression of genes involved in inflammation, adhesion and invasion. KSHV-encoded latent protein vFLIP K13 has been previously shown to promote the survival and proliferation of PEL cells. In this study, we employed gene array analysis to characterize the effect of K13 on global gene expression in PEL-derived BCBL1 cells, which express negligible K13 endogenously. We demonstrate that K13 upregulates the expression of a number of NF-κB responsive genes involved in cytokine signaling, cell death, adhesion, inflammation and immune response, including two NF-κB subunits involved in the alternate NF-κB pathway, RELB and NFKB2. In contrast, CD19, a B cell marker, was one of the genes downregulated by K13. A comparison with K13-induced genes in human vascular endothelial cells revealed that although there was a considerable overlap among the genes induced by K13 in the two cell types, chemokines genes were preferentially induced in HUVEC with few exceptions, such as RANTES/CCL5, which was induced in both cell types. Functional studies confirmed that K13 activated the RANTES/CCL5 promoter through the NF-κB pathway. Taken collectively, our results suggest that K13 may contribute to the unique gene expression profile, immunophenotype and clinical presentation that are characteristics of KSHV-associated PEL.

## Introduction

Kaposi sarcoma-associated herpesvirus (KSHV) is directly associated with Kaposi sarcoma and several lymphoproliferative disorders (LPDs) including primary effusion lymphoma (PEL) and multicentric Castleman's disease (MCD) [1]. PEL is a highly malignant plasmablastic tumor that most frequently affects body cavities such as the pericardial or pleural spaces [1]. This clonal B cell tumor is characterized by the lack of expression of most B and T cell markers and thus has a “null phenotype” [1]. PEL cells over-express genes involved in inflammation, cell adhesion and evasion, which is believed to contribute to their unique presentation in body cavities [2].

A hallmark of all herpesviruses is their ability to establish a lifelong latent infection. Five major KSHV proteins are present in the cells latently infected with the virus, including LANA (Latency associated nuclear antigen), vCyclin, vFLIP (viral FLICE inhibitory protein, also called K13), vIL6, and vIRF3 [3], [4]. LANA, vCyclin and vFLIP K13 are transcribed from the same genomic region into a single tricistronic mRNA, which gets alternatively spliced into three transcripts [5]. The K13 gene encodes for a protein with homology to the prodomain of caspase 8/FLICE [6]. The K13 protein was originally thought to protect KSHV-infected cells from apoptosis by preventing the activation of caspase 8/FLICE and, as such, was classified as a viral FLICE inhibitory protein (vFLIP) [6]. However, subsequent work from our laboratory and others showed that K13 is a potent activator of the NF-κB pathway and manipulates this pathway to promote cellular survival, proliferation, transformation and cytokine secretion [7], [8], [9], [10], [11], [12], [13], [14], [15].

To understand how viruses subvert host molecular pathways and cause cellular transformation has been a fascinating and challenging task. The advent of microarray technology has made it feasible to perform whole genome expression profiling of different disease states [16]. In the conventional method microarray data analysis, only the top few individual genes that are highly differentially expressed between two phenotypes are analyzed [16]. Although such individual genes may prove to be relevant for KSHV infection, it is increasingly doubted whether large fold changes in individual genes have more biological relevance as compared to smaller but coordinated fold changes in a set of genes encoding proteins that belong to a single pathway [17]. As in biological processes, genes often cooperate in the so-called biological pathways, and therefore analyzing microarray data at the level of pathways might yield better insights into biological mechanisms associated with the pathogenesis of a particular disease [18]. In addition, integrating genes into functional sets allow consideration of all genomic information available from a microarray platform rather than focusing on individual genes passing a certain significance threshold [17], [19], [20].

Previous work from our laboratory and others has shown that ectopic expression of K13 in human umbilical vein endothelial cells (HUVECs) induces them to acquire a spindle cell phenotype, which is accompanied by exuberant production of pro-inflammatory cytokines and chemokines known to be involved in the pathogenesis of KS lesions [15], [21]. Using gene expression analysis, we further demonstrated that K13 may account for change in the expression of a significant proportion of genes following KSHV infection of vascular endothelial cells [22]. Although KSHV is also associated with PEL and MCD very little is known about the effect of K13 on gene expression in lymphoid cells. As such, in this study, we have studied the effect of ectopic K13 expression on gene expression and activation of signaling pathways in PEL-derived BCBL1 cells, which express negligible K13 endogenously. Furthermore, to determine whether K13 affects gene expression differently between lymphoma and vascular endothelial cells, we have compared our newly generated dataset of BCBL1 with our publicly available microarray expression dataset of K13-expressing HUVECs.

## Materials and Methods

### Cell lines and reagents

293T and BCBL1 cells were obtained from the American Type Culture Collection. The IL-6 dependent murine T1165 plasmacytoma cell line has been described earlier [23]. Polyclonal populations of BCBL1 cells expressing K13 and K13-ER^TAM^ have been described previously [21], [24]. FLAG antibody was purchased from Sigma and mouse 8F6 monoclonal antibody against full length K13 was generated in our lab as detailed previously [25]. NF-κB inhibitors Bay-11-7082, IKK inhibitor VI and PS-1145 were purchased from Calbiochem (San Diego, CA) and Arsenic trioxide (As_2_O_3_) was from Sigma-Aldrich (St. Louis, MO).

### Gene chip human array

We used the human genome HGU-133 plus 2.0 arrays (Affymetrix, Santa Clara, CA); an oligonucleotide-probe based gene array chip containing ∼50,000 transcripts, which provides a comprehensive coverage of the whole human genome.

### RNA isolation and hybridization to Gene arrays

BCBL1 cells stably expressing empty vector MSCV or MSCV K13-ER^TAM^-encoding constructs were treated with 4OHT (20 nM) or solvent for 48 h. Total RNA was isolated using Qiagen RNeasy kit (Qiagen, Valencia, CA). Ten micrograms of total RNA was used to synthesize cDNA. T7 promoter introduced during the first strand synthesis was then used to direct cRNA synthesis, which was labeled with biotinylated deoxynucleotide triphosphate, following the manufacturer's protocol (Affymetrix, San Diego, CA). After fragmentation, the biotinylated cRNA was hybridized to the gene chip array at 45°C for 16 h. The chip was washed, stained with phycoerytherin-streptavidin, and scanned with the Gene Chip Scanner 3000. After background correction, data analysis was done using PLIER16 (probe logarithmic intensity error) algorithm using Gene Spring GX11.0 (Agilent Technologies, Santa Clara, CA). The microarray data has been deposited with NCBI GEO database (GSE37355).

### Luciferase reporter assay

A luciferase reporter plasmid containing the RANTES promoter was kindly provided by Dr. Robert Schleliner (Northwestern University). Expression constructs for K13 and K13-58AAA and phosphorylation-resistant mutants of IκBα have been described previously [7], [26]. 293T cells were transfected in a 24-well plate with various test plasmids along with the RANTES luciferase reporter constructs (75 ng/well) and a pRSV/LacZ (â??-galactosidase) reporter construct (75 ng/well), as described previously [27]. Cells were lysed 24-36 hours later, and cell extracts were used to measure firefly luciferase and â-galactosidase activities, respectively. Luciferase activity was normalized by the â-galactosidase activity to control for differences in transfection efficiency.

### Gene set enrichment analysis (GSEA)

Nonparametric gene set enrichment analysis (GSEA) was performed using GSEA 2.0 (Broad Institute, Cambridge, MA) [19]. This method ranks genes according to their relative difference in expression (Student's *t*-statistic) between two phenotypes of K13-ER^TAM^ cell (with and without 4OHT treatment). GSEA compares this ranked list of genes to a large collection of pathway data gene sets and assigns an enrichment score, if gene is present in the dataset its score is increased and if it is absent the score is decreased. The enrichment statistics is the maximum deviation of running enrichment score from zero. The gene sets that significantly perform the random-class permutations are considered significant. A significance threshold was set at a nominal *p*-value of 0.05 and a false discovery rate (FDR)<0.20, which is the estimated probability that a gene set with a given enrichment statistic represents a false-positive finding. The gene set with an FDR<0.20 indicates that the result is likely to be valid 8 out of 10 times.

### Analysis of enrichment of Transcription Factor Binding sites (TFBS)

Transcription factor (TF) binding sites show the highest density around the transcription start site (TSS); therefore we restricted our analysis to 1000 bp upstream and downstream of TSS site. We used Biomart tool of Ensembl to retrieve 5′ flanking regions of each transcript. The conserved non-coding regions of the promoters were searched for matches to all TFBS profiles both in TRANSFAC [28] and JASPAR databases [29]. Briefly, while searching both databases in TELiS (Transcription Element Listening System), the parameters were set in order to set an FDR<20% (TRANSFEC) or <10**%** (JASPER) with corresponding p<0.01. We also validated the JASPAR and TRANSFAC predicted transcription factor binding sites in Matinspecter of Genomatrix (Genomatrix, Munchen) as well as oPossum data bases [30].

### Real-time PCR

cDNA was synthesized from RNA samples by PCR RNA core kit (Applied Biosystems, Bedford, MA). Real time quantitative reverse transcript-polymerase chain reaction (qRT-PCR) was performed with SYBER Green, using gene-specific PCR primers listed in Table S1. Samples were run in triplicate, and PCR was performed by an ABI Step One Plus thermocycler (Applied Biosystems, Bedford, MA). To take into consideration any change in the reference housekeeping gene, we used 4 representative reference genes (GNBL, β-2-microglobulin, GAPDH and 18S RNA) to calculate normalization factor (NF) using geNorm [31] module in qbase^PLUS^software [32]. In addition, the efficiency (E) of PCR in each run was also determined. Both NF and E were used to report relative expression of the gene of interest using 2^−**ΔΔ**^ ct method as detailed earlier [33]. The statistical significance of expression was calculated by two sided paired t-test and Pearson Correlation coefficient between gene expression array and real time PCR was calculated using R statistical software.

### Cell viability and cell-cycle assays

To study the biological activity of K13-induced IL6 secretion, the supernatants from untreated and 4OHT-treated BCBL1 cells expressing vector and K13-ER^TAM^ cells were analyzed for the growth of IL-6 dependent T1165 cells. T1165 cells from exponentially growing cultures were washed three times with hIL6-free medium and plated in a flat-bottom 96-well plate at a density of 5×10^3^ cells/well in the presence or absence of BCBL1 supernatants or hIL6. Cell viability was measured after 48 hours using the MTS reagent (3-4,5-dimethylthiazol-2yl)-5-(3-carboxy-methoxyphenyl)-2-(4-sulfophenyl)-2H-tetrazolium, inner salt) following manufacturer's instructions (Promega, Madison, WI). Absorbance of viable cells was measured at 490 nm with 600 nm as a reference wavelength. Percent cell survival was calculated based on the reading of cells grown in the presence of hIL6 as 100%. DNA content analysis was performed as described previously [7]. Briefly, cell pellets were fixed in 70% ethanol, and incubated at 4°C overnight. For staining, the cell pellets were re-suspended in 0.5 ml of 0.05 mg/ml Propidium Iodide (PI) plus 0.2 mg/ml RNAseA and incubated at 37°C for 30 minutes. Cell cycle distribution was analyzed on a BD Biosciences LSR II flow cytometry instrument.

### Enzyme linked Immunosorbent assay (ELISA)

Human CCL5 (RANTES) was measured in the cell culture supernatant using a CCL5-ELISA kit from R&D systems (Minneapolis, MN) and following the recommendations of the manufacturer.

## Results

### Differential gene expression levels in BCBL1 -K13 cells

To study the impact of K13 on gene expression in PEL cells, we took advantage of the PEL-derived BCBL1 cells as they express negligible amount of K13 endogenously [24]. We had previously generated BCBL1 cells stably expressing a K13-ER^TAM^ fusion construct in which K13 is fused in-frame to the ligand -binding domain of a mutated estrogen receptor [24]. The mutated estrogen receptor does not bind to its physiological ligand estrogen, but binds with very high affinity to the synthetic ligand 4OHT (4-hydroxytamoxifen) and regulates the activity of K13 in a 4OHT-dependent manner. The BCBL1 cells expressing either mock vector or K13-ER^TAM^ were treated with 4OHT for 48 hours and RNA was harvested. High quality RNA was used to run gene expression array using Affymetrix HG-U133 plus 2 chip representing more than 50,000 annotated transcripts. Analysis of normalized fluorescence intensities indicated that in the cells expressing empty vector, expression ratios of most of the genes with and without 4OHT treatment remained close to 1 or changed slightly (≤2), suggesting that 4OHT by itself does not have any significant effect on gene expression. In contrast, induction of K13-ER^TAM^ expression by 4OHT treatment changed the expression ratio of a significant proportion of genes more than 2 fold (data not shown). Next, the gene array data was uploaded to Gene Spring GX11.0 software, and after median shift normalization the primary analysis was performed using PLIER16 algorithm, as recommended in the workflow of the software. Table 1 enlists top 50 upregulated and 15 downregulated genes. Among the genes upregulated by K13 were several known NF-κB-responsive genes, such as BIRC3, TNFAIP3, EBI3, NFKBIA, FAS, RELB and NFKB2. In contrast, CD19, a B cell marker, was among the genes whose expression was down-regulated by K13. A complete list of genes differentially expressed in 4OHT-treated BCBL1-K13-ER^TAM^ cells is provided as Table S2.

**Table 1:** Summary of differentially regulated gene clusters in 4OHT-treated K13-ER^TAM^-transduced BCBL1 cells.

*S.No.*	*Entrez Gene*	*Gene Symbol*	*Gene Title*	*RefSeq Transcript ID*	*Fold change*	*Regulation*
1.	10537	UBD	ubiquitin D	NM_001470	26.07	Up
2.	5328	PLAU	plasminogen activator, urokinase	NM_001145031	19.66	Up
3.	5645	PRSS2	protease, serine, 2 (trypsin 2)	NM_002770	14.84	Up
4.	330	BIRC3	baculoviral IAP repeat-containing 3	NM_001165	13.51	Up
5.	5644	PRSS1	protease, serine, 1 (trypsin 1)	NM_002769	13.24	Up
6.	4050	LTB	lymphotoxin beta (TNF superfamily, member 3)	NM_002341	12.98	Up
7.	7128	TNFAIP3	tumor necrosis factor, alpha-induced protein 3	NM_006290	12.85	Up
8.	154754	PRSS1	protease, serine, 1 (trypsin 1)	NM_002769	12.56	Up
9.	7262	PHLDA2	pleckstrin homology-like domain, family A, member 2	NM_003311	10.51	Up
10.	10148	EBI3	Epstein-Barr virus induced 3	NM_005755	9.90	Up
11.	4914	NTRK1	neurotrophic tyrosine kinase, receptor, type 1	NM_001007792	9.51	Up
12.	6352	CCL5	chemokine (C-C motif) ligand 5	NM_002985	9.42	Up
13.	4792	NFKBIA	nuclear factor of kappa light polypeptide gene enhancer in B-cells inhibitor, alpha	NM_020529	8.33	Up
14.	2015	EMR1	egf-like module containing, mucin-like, hormone receptor-like 1	NM_001974	8.10	Up
15.	355	FAS	Fas (TNF receptor superfamily, member 6)	NM_000043	7.94	Up
16.	972	CD74	CD74 molecule, major histocompatibility complex, class II invariant chain	NM_001025158	7.02	Up
17.	3117	HLA-DQA1	major histocompatibility complex, class II, DQ alpha 1	NM_002122	6.73	Up
18.	259307	IL4I1	interleukin 4 induced 1	NM_152899	6.58	Up
19.	51700	CYB5R2	cytochrome b5 reductase 2	NM_016229	6.44	Up
20.	1543	CYP1A1	cytochrome P450, family 1, subfamily A, polypeptide 1	NM_000499	6.01	Up
21.	25907	TMEM158	transmembrane protein 158	NM_015444	5.81	Up
22.	7273	TTN	Titin	NM_003319	5.21	Up
23.	84033	OBSCN	obscurin, cytoskeletal calmodulin and titin-interacting RhoGEF	NM_001098623	5.17	Up
24.	7412	VCAM1	vascular cell adhesion molecule 1	NM_001078	5.07	Up
25.	9294	S1PR2	sphingosine-1-phosphate receptor 2	NM_004230	5.03	Up
26.	5971	RELB	v-rel reticuloendotheliosis viral oncogene homolog B	NM_006509	4.97	Up
27.	5996	RGS1	regulator of G-protein signaling 1	NM_002922	4.97	Up
28.	9308	CD83	CD83 molecule	NM_001040280	4.94	Up
29.	3383	ICAM1	intercellular adhesion molecule 1	NM_000201	4.93	Up
30.	25801	GCA	grancalcin, EF-hand calcium binding protein	NM_012198	4.92	Up
31.	9235	IL32	interleukin 32	NM_001012631	4.81	Up
32.	8651	SOCS1	suppressor of cytokine signaling 1	NM_003745	4.75	up
33.	11226	GALNT6	UDP-N-acetyl-alpha-D-galactosamine:polypeptide N-acetylgalactosaminyl- transferase 6	NM_007210	4.70	Up
34.	1999	ELF3	E74-like factor 3 (ets domain transcription factor, epithelial-specific)	NM_001114309	4.68	Up
35.	3109	HLA-DMB	major histocompatibility complex, class II, DM beta	NM_002118	4.50	Up
36.	115019	SLC26A9	solute carrier family 26, member 9	NM_001142600	4.49	Up
37.	3561	IL2RG	interleukin 2 receptor, gamma (severe combined immunodeficiency)	NM_000206	4.48	Up
38.	4261	CIITA	class II, major histocompatibility complex, transactivator	NM_000246	4.34	Up
39.	4791	NFKB2	nuclear factor of kappa light polypeptide gene enhancer in B-cells 2 (p49/p100)	NM_001077493	4.29	Up
40.	718	C3	complement component 3	NM_000064	4.29	Up
41.	285025	CCDC141	coiled-coil domain containing 141	NM_173648	4.29	Up
42.	100132999	LOC100132999	hypothetical protein LOC100132999	XM_001722799	4.16	Up
43.	6892	TAPBP	TAP binding protein (tapasin)	NM_003190	4.14	Up
44.	6236	RRAD	Ras-related associated with diabetes	NM_001128850	4.12	Up
45.	27074	LAMP3	lysosomal-associated membrane protein 3	NM_014398	3.99	Up
46.	2232	FDXR	ferredoxin reductase	NM_004110	3.98	Up
47.	3119	HLA-DQB1	major histocompatibility complex, class II, DQ beta 1	NM_002123	3.77	Up
48.	114294	LACTB	lactamase, beta	NM_032857	3.70	Up
49.	64319	FBRS	Fibrosin	NM_001105079	3.70	Up
50.	780	DDR1	discoidin domain receptor tyrosine kinase 1	NM_001954	3.68	Up
51.	11124	FAF1	Fas (TNFRSF6) associated factor 1	NM_007051	3.31	Down
52.	55916	NXT2	nuclear transport factor 2-like export factor 2	NM_018698	2.74	Down
53.	51616	TAF9B	TAF9B RNA polymerase II, TATA box binding protein (TBP)-associated factor	NM_015975	2.71	Down
54.	2162	F13A1	coagulation factor XIII, A1 polypeptide	NM_000129	2.63	Down
55.	3753	KCNE1	potassium voltage-gated channel, Isk-related family, member 1	NM_000219	2.53	Down
56.	55737	VPS35	vacuolar protein sorting 35 homolog (S. cerevisiae)	NM_018206	2.45	Down
57.	51474	LIMA1	LIM domain and actin binding 1	NM_001113546	2.43	Down
58.	135932	TMEM139	transmembrane protein 139	NM_153345	2.38	Down
59.	2844	GPR21	G protein-coupled receptor 21	NM_005294	2.34	Down
60.	930	CD19	CD19 molecule	NM_001770	2.32	Down
61.	6711	SPTBN1	spectrin, beta, non-erythrocytic 1	NM_003128	2.30	Down
62.	2744	GLS	Glutaminase	NM_014905	2.29	Down
63.	3890	KRT84	keratin 84	NM_033045	2.23	Down
64.	54704	PPM2C	protein phosphatase 2C, magnesium-dependent, catalytic subunit	NM_018444	2.23	Down
65.	1131	CHRM3	cholinergic receptor, muscarinic 3	NM_000740	2.22	Down

### Pathway enrichment in K13 expressing cells

We used a unique approach of defining the various biological pathways affected by K13, where expression ratios of all the genes in the dataset were used to divide genes in various sets in terms of their biological relatedness. Gene set enrichment analysis (GSEA) using pathway definitions from Biocarta gene sets database revealed 9 significant gene sets specifically affected by K13 expression at a nominal *p*-value<0.05 and FDR<0.20. There are several well-known pathways affecting the cellular growth and cancer progression in these gene sets, such as Cytokine, Death, NF-κB and Inflammatory pathways (Table 2). In a complementary approach, GSEA using pathway definitions from Kyoto Encyclopedia of Genes and Genomes (KEGG) database revealed 10 gene sets significantly up-regulated in K13-expressing BCBL1 cells at a nominal *p*-value<0.05 and FDR<0.20. This analysis also identified several pathways linked to immune and inflammatory response and cell signaling, such as antigen processing and presentation, apoptosis, adipo-cytokine signaling, Toll like receptor signaling, cell adhesion molecules, epithelial cell signaling and cytokine-cytokine receptor interaction pathways (Table 2). Thus, despite the difference in definition of gene sets using the KEGG and Biocarta databases, the pathways identified to distinguish cellular responses attributed to K13 expression were comparable. These well accepted and widely used databases not only differ in their pathway definitions, but also offer different analyzable gene sets. For example, the newly discovered HIV-NEF, NKT and neutrophil-controlling FMLP pathways were not present, as such, in the KEGG database, which could probably explain why they were identified only in the GSEA using the Biocarta database.

**Table 2:** Gene sets enriched in BCBL-K13 and HUVEC-K13 cells (a) Biocarta gene sets, (b) KEGG (Kyoto Encyclopedia of Genes and Genomes) gene sets.

*S.No.*	*Gene Set*	*No. of genes in set*	*ES*	*NES*	*NOMp-value*	*FDR q-value*	*RANKAT MAX*
*BCBL-K13 (a)-Biocarta*							
1	Cytokine pathway	20	−0.70	−1.82	0.007	0.087	1546
2	Death pathway	33	−0.62	−1.79	0.000	0.066	1824
3	NF-κB pathway	23	−0.68	−1.76	0.005	0.062	1824
4	HIV Nef pathway	55	−0.55	−1.74	0.002	0066	2079
5	Inflammatory pathway	29	−0.61	−1.71	0.005	0.072	1546
6	NKT pathway	26	−0.57	−1.53	0.042	0.271	1709
7	FMLP pathway	35	−0.52	−1.53	0.028	0.237	2728
8	Stress pathway	24	−0.56	−1.51	0.025	0.240	3078
9	NTHI pathway	21	−0.59	−1.50	0.049	0.227	2824
*BCBL-K13 (b)-KEGG*							
1	Antigen processing and presentation	75	−0.62	−2.06	0.000	0.002	1538
2	Diabetes mellitus	41	−0.65	−1.92	0.000	0.010	2777
3	Apoptosis	83	−0.57	−1.89	0.000	0.011	2661
4	Adipocytokine signaling pathway	72	−0.56	−1.86	0.000	0.015	3555
5	Toll like receptor signaling pathway	100	−0.51	−1.76	0.000	0.041	2889
6	Cell adhesion molecules	125	−0.49	−1.74	0.000	0.044	3225
7	Small cell lung cancer	87	−0.50	−1.69	0.003	0.062	2889
8	Thyroid cancer	29	−0.59	−1.62	0.014	0.107	3555
9	Epithelial cell signaling	65	−0.47	−1.54	0.009	0.200	560
10	Cytokine cytokine receptor interaction	244	−0.40	−1.53	0.001	0.196	1950
*HUVEC-K13 (a)-Biocarta*							
1	Inflammatory pathway	29	−0.86	−2.21	0.000	0.000	486
2	Cytokine pathway	20	−0.83	−1.99	0.000	0.003	138
3	IL-1Receptor pathway	31	−0.74	−1.99	0.002	0.002	959
4	NF-κB pathway	23	−0.80	−1.99	0.000	0.002	959
5	DC pathway	21	−0.78	−1.92	0.002	0.008	224
6	Toll pathway	33	−0.68	−1.81	0.006	0.029	1298
7	NTHI pathway	21	−0.76	−1.80	0.002	0.029	959
8	Death pathway	33	−0.66	−1.80	0.002	0.026	1712
9	HIV NEF pathway	55	−0.61	−1.78	0.002	0.029	2101
10	NKT pathway	26	−0.69	−1.75	0.013	0.036	800
11	IL-6 pathway	21	−0.70	−1.72	0.011	0.042	1766
12	IL-12 pathway	20	−0.68	−1.63	0.037	0.088	1446
13	Raccycd pathway	22	−0.62	−1.59	0.045	0.112	928
14	Stress pathway	24	−0.60	−1.51	0.074	0.180	949
*HUVEC-K13 (b)-KEGG*							
1	Cytokine cytokine receptor interaction	244	−0.73	−2.69	0.000	0.000	863
2	Antigen processing and presentation	75	−0.80	−2.48	0.000	0.000	1246
3	Toll like receptor signaling pathway	100	−0.74	−2.38	0.000	0.000	1053
4	Epithelial cell signaling	65	−0.65	−2.01	0.000	0.002	1170
5	Diabetes mellitus	41	−0.73	−2.00	0.000	0.001	1192
6	JAK STAT pathway	151	−0.58	−1.99	0.000	0.001	1766
7	Cell adhesion molecules	125	−0.59	−1.99	0.000	0.001	1025
8	Hematopoietic cell lineage	84	−0.61	−1.93	0.000	0.003	713
9	Adipocytokine signaling pathway	72	−0.60	−1.84	0.000	0.010	1766
10	Small cell lung cancer	87	−0.58	−1.83	0.000	0.010	928
11	Aminosugars metabolism	29	−0.66	−1.69	0.019	0.051	1425
12	Apoptosis	83	−0.54	−1.69	0.004	0.047	1161
13	Natural killer cell mediated cytotoxicity	127	−0.49	−1.65	0.004	0.062	2245
14	T cell receptor signaling pathway	93	−0.49	−1.59	0.004	0.101	1755

### Comparative Analysis of K13 induced genes in BCBL1 and HUVEC cells

**Figure 1:**
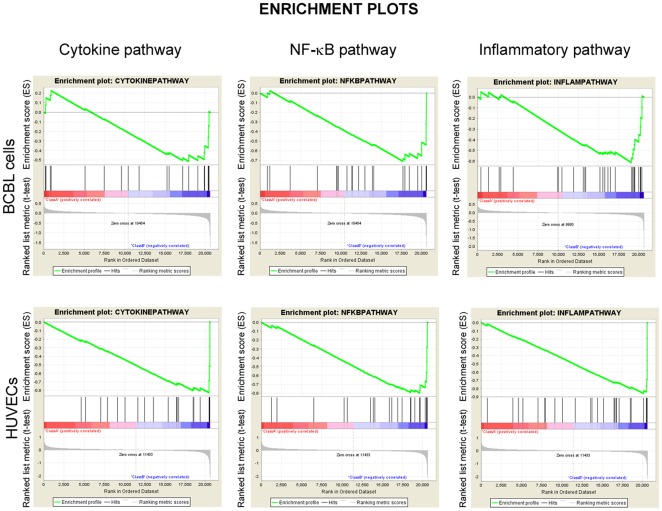
Gene set enrichment analysis. For Gene set enrichment analysis of signatures genes from BCBL1-K13 (top panel) and HUVEC-K13 (lower panel), the t-test was graphed for each correlated gene in the ranked dataset. Three GSEA enrichment plots for representative biological pathways (Cytokine, NF-κB and Inflammatory) enriched in 4OHT-treated BCBL1-K13-ER^TAM^ and HUVEC-K13-ER^TAM^ are shown. The top portion of each GSEA plot shows the running enrichment score for validated genes specific for particular pathway as it moves down the ranked list. The bottom portion of each plot shows the value of ranking matrices as it moves down the list of ranked genes. The red horizontal bar which terminate with blue color indicate shift from positively correlated genes (red) to negatively correlated genes (blue). Further detailed interpretation about these plots can be found at Broad Institute web site (http://www.broadinstitute.org/gsea/index.jsp).

We have previously generated K13-ER^TAM^ expressing HUVEC and used them to study the effect of K13 on gene expression. We compared our newly generated gene expression data set of K13-ER^TAM^ expressing BCBL1 with our publically available gene expression data set of K13-ER^TAM^ expressing HUVEC (GSE16051) to determine if K13 affects genes differentially in different cell lineages, which could explain, in part, the distinct clinical presentations of clinical disorders associated with KSHV infection. Among the genes showing >2 fold induction following treatment with 4OHT, there were 42 genes that were shared between the BCBL1 and HUVEC data sets (Table S3). For example, Ubiquitin D was the most upregulated gene in 4OHT-treated BCBL1-K13-ER^TAM^ cells and the second most upregulated gene in 4OHT treated HUVEC-K13-ER^TAM^ cells. Additionally, genes belonging to the NF-κB, Cytokine and Inflammatory, HIV-NEF and NKT pathways were enriched in both datasets. Representative enrichment plots showing t-test for each correlated gene in the ranked dataset for Cytokine, NF-κB and inflammatory pathways are shown in Figure 1.

Although there was an overlap in the pathways activated by K13 in BCBL1 and HUVEC cells, there was a significant difference in the types and magnitude (fold induction) of genes induced by K13 in the two cell types. Thus, 4OHT treatment resulted in >20 fold induction of 14 genes in HUVEC-K13-ER^TAM^ cells (Table S4). In contrast, only one gene, Ubiquitin D, was induced >20 fold in 4OHT-treated BCBL1-K13-ER^TAM^ cells (26.07 fold induction) (Table 1). Interestingly, although ubiquitin D was the most upregulated gene in BCBL1-K13-ER^TAM^ cells and the second most upregulated gene in HUVEC-K13-ER^TAM^ cells, there was a significant difference in the magnitude of induction (26.07 fold *vs* 61.02 fold) for this gene in the two cell types. Furthermore, a majority of genes whose expression was strongly induced by K13 in HUVECs were chemokines such as CCL2 (73.04 fold), CCL20 (63.8 fold), CCL5 (49.7 fold), CXCL10 (53.32 fold), IL8 (18.4 fold), CX3CL1 (30.48 fold) and IL6 (17.57 fold) (Table S4). In contrast, CCL5 (9.4 fold) and IL32 (4.8 fold) were the only chemokines whose expression was upregulated greater than 4-fold by K13 in BCBL1 cells (Table 1). Interestingly, suppressor of cytokine signaling 1 (SOCS1), a gene known to be involved in dampening of inflammatory response was upregulated 4.75 fold by K13 in BCBL1 cells but not in HUVECs (Table S4). Taken collectively, the above results demonstrate that although K13 activates the NF-??êB pathway in both BCBL1 cells and HUVEC, it affects gene expression differentially in the two cell lineages and strongly upregulates genes belonging to proinflammatory chemokines mainly in HUVECs.

### Transcriptional control of K13-induced gene expression

It has been well established that genes with common transcription factor (TF) binding sites have higher likelihood of sharing similar expression profiles [34], [35]. Indeed, it is a widely accepted hypothesis that genes that are co-expressed share common regulatory motifs [36], [37], [38]. Therefore, we next examined if genes induced by 4OHT treatment in K13-ER^TAM^ expressing BCBL1 and HUVECs were coordinately regulated by common TFs. For this purpose, we searched the JASPER database for TFs with binding sites over-represented in promoters of genes upregulated by K13 in BCBL1 and HUVECs. In the newly generated gene expression data set of 4OHT-treated BCBL1 K13-ER^TAM^ -expressing cells, there were 6 transcription factors whose binding sites were significantly over-represented in the promoters of K13-induced genes according to the JASPER database (p<0.01, FDR<0.10) (Table 3). Among these, three belonged to the members of the NF-κB/REL family. To confirm the results, we also searched the TRANSFAC database and similarly found over-representation of binding sites for the members of the NF-κB/REL family among the promoters of the genes induced by K13 in BCBL1 cells. We next repeated the analysis with our previously published HUVEC dataset. There were 10 sites that were over-represented in JASPAR database using stringent parameters (p<0.01, FDR<0.10), while 14 binding sites were over-represented in TRANSFAC (p<0.01, FDR<0.15) database. Similar to the BCBL1 dataset, NF-κB/REL binding sites were again over-represented among the promoters of the genes induced by K13 in HUVEC in both databases. Thus, consistent with our published studies [9], [27] and the results of the pathway analysis, NF-κB/REL is the major transcription factor responsible for gene induction by K13 in both BCBL1 and HUVECs. However, in addition to NF-κB, several other TF binding sites were also over-represented among the promoters of genes upregulated by K13.

**Table 3:** Transcription factors with binding sites over-represented in promoters of genes upregulated by K13 in BCBL1 and HUVECs.

*JASPAR database in BCBL1-K13 cells*				
*S.No.*	*Sequence motif*	*Description*	*p- value*	*% of input*
1	Jaspar NF-kappaB	REL	2.16E-6	29.6
2	Jaspar p65	REL	0.0001	20.1
3	Jaspear Irf-1	TRP-cluster	0.0025	17.6
4	Jaspar CFI-USP	Nuclear receptor	0.0027	16.4
5	Jaspar p50	REL	0.001	15.1
6	Jaspar COUP-TF	Nuclear receptor	0.0030	4.4
7	Jaspar Irf-2	TRP-cluster	0.0088	2.6

*TRANSFEC database in BCBL1-K13 cells*				
1	V$NFKAPPAB-01: NF-kappaB	NF-kappaB	1.07E-5	27.7
2	V$NFKAPPAB65-01: NF-kappaB	NF-kappaB (p65)	3.16E-5	24.5
3	V$NFKAPPAB50-01: NF-kappaB	NF-kappaB (p50)	0.0002	17
4	V$NFKB-Q6: NF-kappaB	NF-kappaB	0.0001	12
5	V$IK3-01: Ik-3	Ikaros 3	0.0012	8.8
6	V$COUP-01: COUP-TF	COUP/HNF-4 heterodimer	0.0031	3.0
7	V$PAX2-01: Pax-2	Pax-2	0.0024	2.6
8	V$NRSF-01: NRSF	neuron-restrictive silencer factor	9.56E-6	1.2

*JASPAR database in HUVEC-K13*				
1	Jaspar c-REL	REL	1.00E-10	37.8
2	Jaspar NF-kappaB	REL	3.99E-6	32.7
3	Jaspar Irf-1	TRP-Cluster	1.00E-10	26.5
4	Jaspar p65	REL	1.00E-10	22.5
5	Jaspar Dorsal-2	REL	4.84E-6	20.4
6	Jaspar p50	REL	0.0006	19.4
7	Jaspar c-FOS	bZIP	0.0004	14.3
8	Jaspar Irf-2	TRP-Cluster	1.00E-10	11.2
9	Jaspar Brachyury	T-BOX	0.0006	1.2
10	Jaspar COUP-TF	Nuclear receptor	0.0088	1.1

*TRANSFEC database in HUVEC-K13*				
1	V$NFKAPPAB-01: NF-kappaB	NF-kappaB	2.19E-6	38.7
2	V$CREL-01: c-Rel	c-Rel	0.0003	37.8
3	V$AP1-C: AP-1	AP-1 binding site	0.0008	37.7
4	V$STAT-01: STATx	signal transducers and activators of transcription	0.0003	32.8
5	V$NFKAPPAB65-01: NF-kappaB	NF-kappaB (p65)	1.00E-10	28.6
6	V$NFKB-C: NF-kappaB	NF-kappaB binding site	1.00E-10	19.4
7	V$NFKAPPAB50-01: NF-kappaB	NF-kappaB (p50)	0.0005	19.4
8	V$NFKB-Q6: NF-kappaB	NF-kappaB	1.00E-10	18.4
9	V$IRF1-01: IRF-1	interferon regulatory factor 1	1.00E-10	16.3
10	V$IRF2-01: IRF-2	interferon regulatory factor 2	1.53E-6	9.2
11	V$ISRE-01: ISRE	interferon-stimulated response element	0.0022	5.1
12	V$VMAF-01: v-Maf	v-Maf	0.0013	3.1
13	V$TAXCREB-02: Tax/CREB	Tax/CREB complex	1.00E-10	1.1
14	V$MEF2-01: MEF-2	myogenic enhancer factor 2	4.83E-5	1.1

(Genes upregulated more than 1.5-fold were analyzed using the JASPAR and TRANSFEC databases. Results with a p-value of less than 0.05 are shown. % input refers to the number of gene promoters bearing the specific motif compared to total number screened.

### Validation of in silico results

**Figure 2:**
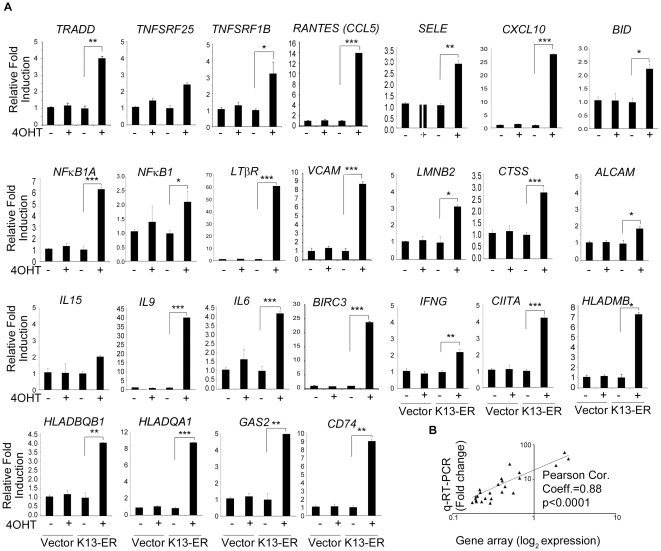
Validation of gene array data by qRT-PCR. (A) Twenty five genes from NF-κB, cytokine, and inflammatory pathways were randomly selected and their relative mRNA levels in mock and 4OHT-treated vector and K13-ER^TAM^-expressing BCBL1 cells were examined using qRT-PCR. Real-time PCR reactions were performed in triplicate and the data presented as fold change mean ±S.E in target gene expression (*p<0.05; Student's t-test). (B) Pearson Correlation coefficient between gene expression array and real time PCR showed a significant agreement (Correlation coefficient 0.88; p<0.0001).

We validated our gene array data by determining mRNA expression of twenty-five highly expressed genes (TRADD, TNFSRF25, TNFSRF1B, RANTES/CCL5, SELE, CXCL10, BID, NFKB1A, NFKB1, LTβR, VCAM, LMNB2, CTSS, ALCAM, IL15, IL9, IL6, BIRC3, CIITA, HLADMB, IFNG, HLADQB1, HLADQ1, GAS2 and CD74) belonging to the cytokine, NF-κB, cell death, cell adhesion and antigen processing pathways (Table S5). We found a good correlation with the expression of the above as determined by real-time RT-PCR and gene array data (Figure 2). As a control for the reliability of qRT-PCR experiments, the mRNA expression of various housekeeping genes such as GAPDH, 18S, GNBL, and β-2-microglobulin was investigated and their expression level in both mock cells as well K13-ER^TAM^ -expressing cells remained unaltered (data not shown).

**Figure 3:**
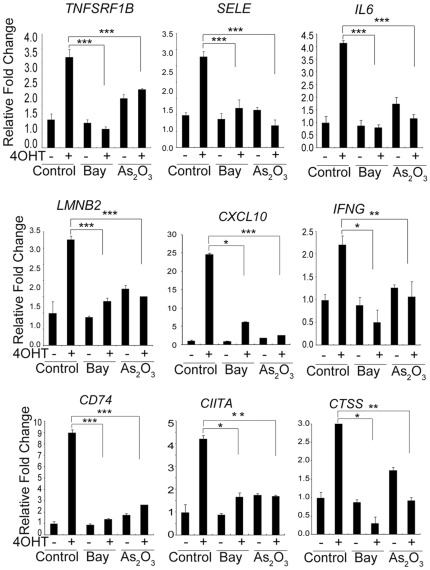
NF-κB inhibitors block K13-regulated genes. BCBL1 K13-ER^TAM^ cells were treated with two NF–κB inhibitors (2 µM Bay 11-7082 or 2 µM As2O3) for 2 hours followed by 4OHT treatment for additional 24 hours and total RNA was isolated as described in the Materials and Method section. Nine genes were randomly picked and their relative mRNA levels were examined using real-time RT-PCR as explained in Figure 2A.

K13-induced NF-κB pathway is known to be effectively blocked by Bay-11-7082 and arsenic trioxide [13], [39]. To examine the role of the NF-κB pathway in the expression of K13-regulated genes in BCBL1 cells, we studied the effect of Bay-11-7082 and arsenic trioxide on 4OHT induced gene induction in BCBL1-K13-ER cells by qRT-PCR analysis. As shown in Figure 3, pretreatment of BCBL1 K13-ER^TAM^ cells with Bay-11-7082 and arsenic trioxide effectively blocked the induction of TNFSRF1B, SELE, CXCL10, NFKB1A, LMNB2, IL6, IFNG, CIITA, CTSS and CD74 genes by 4OHT.

### Mechanism of K13-induced RANTES/CCL5 upregulation

**Figure 4:**
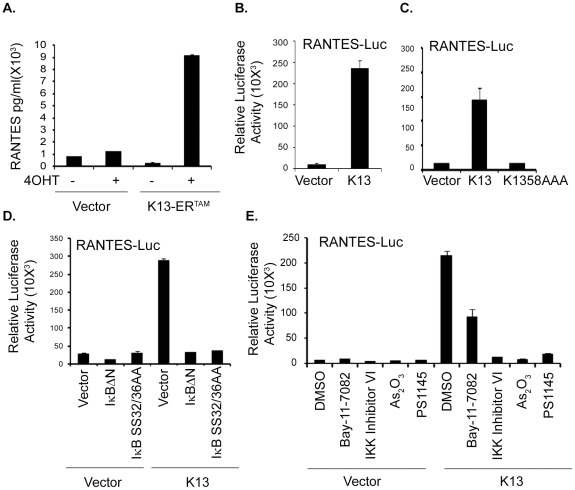
K13-induced NF-κB activity is critical for the activation of RANTES. (A) K13-induced up regulation of RANTES at protein level. Cellular supernatant from BCBL1 vector and K13-ER^TAM^ cells treated with and without 4OHT were collected and used to measure the secretion of RANTES by ELISA. The values shown are averages (mean ± SE) of one representative experiment out of three in which the level of RANTES secretion was measured in triplicate. (B–C) 293T cells were transfected with an empty vector, wild-type K13 (B) or K13-58AAA (C) (250 ng/well) along with a *RANTES* promoter-driven luciferase constructs (75 ng/well) and a pRSV/LacZ (β-galactosidase) reporter construct (75 ng/well), and the reporter assay performed as described under the Materials and Methods section. (D) Dominant-negative mutants of IκBα (IκBαΔN and IκBαSS32/36AA) block K13-induced RANTES promoter activity. 293T cells were transfected either with the indicated plasmids and reporter assay performed as described above. The amount of IκBα mutant plasmids (500 ng/well) was five times the amount of vector or K13 (100 ng/well) plasmid and the total amount of transfected DNA was kept constant by adding empty vector. (E) Pharmacologic inhibitors of NF-κB block K13-induced RANTES promoter activation. 293T cells were transfected with an empty vector or a vector encoding K13 along with RANTES-Luc and pRSV/LacZ reporter constructs. Approximately 3 hours after transfection, cells were treated with dimethyl sulfoxide (vehicle) or the indicated compounds for 18 hours before cell lysis and measurement of reporter activities. Reporter assay was performed as described for Figure 4B–C.

To study the mechanism by which K13 upregulates the expression of genes in BCBL1 cells in greater detail, we selected RANTES/CCL5 as a representative example since its expression was also induced by K13 in HUVECs. RANTES is an important protein involved in immunoregulatory, inflammatory and cell proliferation pathways [40], making its mechanism of upregulation by K13 of biological and clinical significance. We began by validating the results of gene expression and qRT-PCR analyses by checking the effect of K13 on RANTES expression at the protein level. As shown in Figure 4A, we confirmed that expression of RANTES was up-regulated in the supernatant of BCBL1-K13-ER^TAM^ cells upon treatment with 4OHT, as determined by ELISA.

To investigate the mechanism by which K13 upregulates RANTES expression, human embryonic kidney 293T cells were transfected with a luciferase-based reporter construct containing the RANTES gene promoter. K13 strongly activated the RANTES promoter as compared to empty vector-transfected cells (Figure 4B). Since NF-κB pathway was identified as the major pathway induced by K13 by both the pathway analysis and JASPAR/TRANFAC database analysis, we next examined the involvement of this pathway in K13-induced RANTES transcriptional activation. For this purpose, we took advantage of a mutant of K13, K13-58AAA, which lacks the ability to activate the NF-κB pathway [7]. As shown in Figure 4C, K13-58AAA mutant failed to activate the RANTES promoter. Furthermore, K13-induced RANTES activity was effectively blocked by two phosphorylation-resistant mutants of IκBα (IκBα SS32/36AA and IκBαΔN) (Figure 4D–E) that are known to block the NF-κB pathway [27], and by treatment with chemical inhibitors of the NF-κB pathway, including Bay-11-7082 [13], IKK inhibitor VI [41], PS1145 [42] and arsenic trioxide [39]. Collectively, these results confirm the involvement of the NF-κB pathway in K13-induced up-regulation of RANTES.

### Biological consequences of K13-induced gene expression

**Figure 5:**
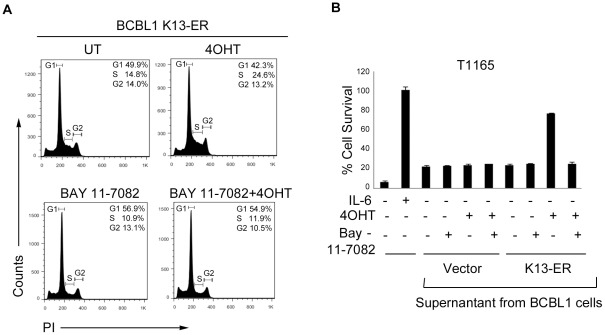
Biological assays to study K13-induced IL6 production and cell cycle analyses. (A) A DNA content analysis showing significant increase in S-phase of cell cycle by 4OHT treatment in serum-starved BCBL1 K13-ER^TAM^ cells and inhibition of this increase in S-phase cells by pre-treatment with Bay-11-7082 (2 µM). DNA content analysis was performed as described previously [7] and explained in Materials and Method section. (B) Cell survival of T1165 cells with supernatants from 4OHT-treated BCBL1-K13-ER^TAM^ cells. BCBL1 vector- and K13-ER^TAM^ cells were pretreated with 2 µM Bay-11-7082 for 2 hours followed by treatment with 4OHT for additional 72 hours and supernatants from these cells were collected and filtered. T1165 cells were treated in triplicate in a 96 well plate (100 µl/well) with 20 µl of cell-free supernatant collected from cells described above. Seventy-two hours post-treatment, cell viability of T1165 cells was measured by MTS assay as described in Materials and Method section. The values (Mean±SEM) shown are from a representative of three independent experiments performed in triplicate.

We have previously shown that ectopic expression of K13 in Rat1 fibroblast cells stimulates cellular proliferation [8] and its shRNA-mediated silencing results in a block in cell proliferation [9]. To study the biological consequences of K13 in PEL, we studied the effect of 4OHT on cell cycle progression in BCBL1-K13-ER^TAM^ cells. As shown in Figure 5A, 4OHT treatment of serum-starved BCBL1-K13-ER^TAM^ cells resulted in an increase in cells in the S-phase as compared to the untreated cells (24.6% *vs* 14.8%), suggesting that K13 stimulates cell-cycle progression from G_1_ to S phase. Treatment with 4OHT had no significant effect on cell cycle progression in the BCBL1-vector cells (data not shown). The stimulatory effect of 4OHT on G_1_ to S phase transition was blocked by treatment with Bay-11-7082, thereby confirming the role of K13-induced NF-κB activation in this process (Figure 5A).

In addition to stimulating the proliferation of KSHV-infected cells, K13 induced cytokine upregulation could contribute to the pathogenesis of KSHV-associated malignancies by stimulating the survival and proliferation of neighboring uninfected cells by acting in a paracrine fashion. To test this hypothesis, we examined the ability of conditioned supernatant from untreated and 4OHT-treated BCBL1-K13-ER^TAM^ cells to support the survival of murine T1165 plasmacytoma cell line that requires IL6 for survival and proliferation [43]. As shown in Figure 5B, survival of T1165 cells was reduced to approximately 6% when grown in IL6-free medium for 72 hours. The addition of conditioned supernatant from untreated or 4OHT-treated BCBL1-vector cells afforded minor protection against IL6-withdrawal-induced cell death (approximately 25% cell survival), reflecting low-level constitutive IL6 secretion from the BCBL1 cells. The conditioned supernatant from untreated BCBL1-K13-ER^TAM^ cells similarly resulted in only partial rescue from IL6-withdrawal-induced cell death (Figure 5B). In contrast, conditioned supernatant from 4OHT-treated BCBL1-K13-ER^TAM^ cells markedly improved the survival (77% survival) of T1165 cells, reflecting upregulation of IL6 secretion by induction of K13 activity (Figure 5B). The survival advantage conferred by the conditioned medium from 4OHT-treated BCBL1-K13-ER^TAM^ cells, however, disappeared if the cells were treated with both 4OHT and Bay-11-7082, confirming a role of K13-induced NF-κB in this process (Figure 5B).

## Discussion

PEL is a rare subset of non-Hodgkin's lymphoma found in patients with HIV/AIDS that predominantly grows in the body cavities as neoplastic effusions, usually without a contiguous tumor mass [44]. Morphologically, PEL shows features that bridge immunoblastic and anaplastic large-cell lymphomas, and frequently displays some degree of plasma cell differentiation [45]. We and others have demonstrated that K13 plays a key role in constitutive NF-κB activity observed in PEL cells [9], [46] and is an oncogenic protein which contribute to lymphoproliferative disorders [11], [47], [48]. In the present investigation, we provide a comprehensive picture of global transcriptional changes induced by K13 in PEL-derived BCBL1 cells. BCBL1 cells are infected with KSHV but express very little K13 endogenously and have negligible constitutive NF-κB activity [24], [49]. As such, we chose BCBL1 cells as a physiological relevant cell line to study the effect of ectopic K13 expression on gene expression in lymphoid cells.

We observed that K13 upregulated the expression of a number of NF-κB-responsive genes in BCBL1 cells. This observation was confirmed by GSEA, qRT-PCR as well as analysis of JASPER and TRANSFAC databases. Nuclear factor-κB (NF-κB) is a critical transcription factor involved in the regulated expression of several genes involved in the inflammatory and immune response [50]. Although many dimeric forms of NF-κB have been described, the classical NF-κB complex is a heterodimer of the p65/RelA and p50 subunits and is found in most cells in association with a family of inhibitory proteins called IκBs [51], [52]. We have previously shown that K13 activates the classical NF-κB pathway by activating a multi-subunit IκB kinase (IKK) complex [53], which contains two catalytic subunits, IKK1/IKKα and IKK2/IKKβ, and a regulatory subunit, NEMO/IKKγ [52], [54]. Activation of the IKK complex by K13 results in phosphorylation of IκB proteins which leads to their ubiquitination and proteasomal-mediated degradation, allowing the classical NF-κB subunits to enter the nucleus and turn on the expression of their target genes [53]. We have also shown that K13 can also activate an alternate NF-κB pathway [9] that involves IKK1/IKKα-mediated phosphorylation of p100/NFKB2 and its slow proteasome-mediated processing into the active p52/p49 subunit that culminates in kinetically slower nuclear translocation of the p52-RelB NF-κB complex [54], [55]. Interestingly, NFKB2/p100 and RelB, the two NF-κB subunits involved in alternate NF-κB pathway, were among the genes upregulated by K13 in BCBL1 cells (Table 1). These results suggest that, in addition to IKK1-induced p100 phosphorylation, transcriptional upregulation of NFKB2 and RelB may also contribute to the activation of the alternate NF-κB pathway by K13.

We also observed significant upregulation of TNFAIP3 (A20) and NFKB1A (IκBα) by K13 in BCBL1 cells. A20 is a deubiquitinating enzyme that is known to act as a tumor suppressor in several subtypes of non-Hodgkin and Hodgkin lymphomas [56]. We have recently demonstrated that A20 blocked K13-induced NF-κB activity and K13-induced upregulation of proinflammatory cytokines in a negative-feedback fashion [49]. A20 was also shown to block K13- induced cellular transformation [49]. In addition to A20, K13 upregulated the expression of NFKB1A, which codes for IκBα, the negative regulator of the classical NF-κB pathway that serves to keep the p65/p50 heterodimers sequestered in an inactive state in the cytoplasm [57]. Finally, the expression of SOCS1 (suppressor of cytokine signaling 1), a negative regulator of the JAK/STAT [58] signaling was significantly induced in the K13-expressing BCBL1 cells. The uncontrolled activation of the NF-κB and JAK/STAT pathways has the potential of resulting in uncontrolled inflammatory response. Therefore, induction of A20, IκBα and SOCS1 by K13 in lymphoid cells may serve to attenuate the inflammatory response and thus help maintain a balance between the virus and the host.

In addition to the positive and negative regulators of the NF-κB pathway, expression of RANTES/CCL5 was significantly upregulated in K13-expressing BCBL1 and HUVECs. Further studies utilizing a RANTES promoter-driven luciferase reporter construct demonstrated that K13 upregulates RANTES through the NF-κB pathway. RANTES is a powerful chemoattractant for blood monocytes, memory T helper cells and eosinophils [59] and as such may contribute to the inflammatory infiltrate present in KSHV-associated malignancies. Additionally, RANTES/CCL5 has been shown to promote tumor proliferation, invasion, metastases and angiogenesis [60] [61], which may also contribute to the development of PEL and KS.

K13 also upregulated the expression of Epstein-Barr virus-induced gene-3 (EBI-3), which associates with p28 to form interleukin-27 (IL-27) or with IL-12 p35 to form IL-35. Both IL-27 and IL-35 have immunosuppressive properties [62]. In particular, IL-35 has been implicated in the suppressive function of regulatory T cells (Treg), which contributes to infection tolerance and tumor progression [62], [63]. Thus, it is conceivable that K13-induced upregulation of EBI-3 may not only contribute to immune tolerance of KSHV-infected cells but also promote progression of PEL and KS via generation of Tregs with suppressive functions.

Another cytokine whose expression was upregulated by K13 in BCBL1 cells was IL-32. A recent study demonstrated that the IL-32 promoter contains NF-κB binding sites [64], which suggests that, similar to the situation with RANTES/CCL5, K13 might upregulate IL-32 through NF-κB activation. Interestingly, there is a good correlation between IL-32 levels and HIV-1 replication in lymphatic tissues and IL-32 was recently shown to play an immunosuppressive role in lymphatic tissues during HIV-1 infection [65]. IL-32 was shown to induce the expression of immunosuppressive molecules IDO (indole-amine-2,3-dioxygenase) and Ig-like transcript 4 in immune cells, which not only block immune activation but also impair host defenses [65]. It is conceivable that the dampening of anti-viral immune response by K13-induced IL-32 induction may enhance HIV-1 replication and persistence, thereby resulting in a synergistic interaction between HIV-1 and KSHV.

A comparative analysis of K13-induced genes in BCBL1 and HUVEC revealed that although K13 primarily induced NF-κB responsive genes in both cell types, there were significant qualitative and quantitative differences. In particular, genes belonging to cytokines and chemokines were highly and differentially induced in HUVEC but not in the BCBL1 cells, with few exceptions, such as RANTES/CCL5 and CXCL10. The differential and robust induction of proinflammatory chemokines and cytokines by K13 in HUVEC may account for the presence of intense inflammatory infiltrate in KSHV associated KS. The underlying reasons for the differential gene induction by K13-induced NF-κB in the two cell types are not clear, but may reflect the presence of lineage-specific positive and negative regulators or epigenetic alterations. It is interesting to note in this regard that SOCS1, a negative regulator of the cytokine signaling, was upregulated only in the K13-expressing BCBL1 cells.

In addition to the NF-κB pathway, genes belonging to a number of other pathways, such as cytokine, death, inflammatory, and antigen processing pathways, were found to be enriched among K13-expressing BCBL1 cells and HUVECs. Similarly, JASPER and TRANSFAC databases revealed the enrichment of a number of transcriptional factors in addition to NF-κB among the promoters of genes upregulated by K13 in BCBL1 and HUVECs. However, there is considerable overlap among the genes induced by the different signaling pathways and transcription factors. In particular, NF-κB pathway is well known for its ability to induce genes belonging to cytokine, death, antigen processing and inflammatory pathways, and to work in concert with other transcription factors, such as AP1 [66]. Therefore, it is conceivable that enrichment of genes belonging to signaling pathways other than the NF-κB pathway may simply reflect this overlap. We also observed that K13 upregulates the expression of a number of chemokines and cytokines, especially in HUVECs, which could work in an autocrine/paracrine fashion to activate a number of secondary signaling pathways and transcription factors, providing an alternative explanation for our results. Studies are in progress to delineate the role of K13 in activation of signaling pathways other than the NF-κB pathway.

PEL display a gene expression profile that is distinct from all non-Hodgkin lymphomas of immunocompetent hosts and AIDS-associated NHL [67]. The gene expression profile of PEL has been defined as plasmablastic as it shares features of both immunoblasts and plasma cells [67]. Interestingly, increased expression of a number of genes found to be induced by K13 in the BCBL1 cells in the present study, including SOCS1, TNFAIP3, NFKB1A, LTB, IL2RG, RELB, RRAD, CCL5, PHLDA2, CIITA, RGS1 and FAS, have been associated with the plasmablastic phenotype in human lymphomas [68]. K13 also upregulated the expression of IL6, a plasma cell growth factor that has been also shown to stimulate the proliferation of PEL cells [69]. PEL cells are typified by their lack of expression of B cell markers [48]. Interestingly, CD19, a B cell marker, was one of the genes downregulated by K13 in BCBL1 cells. Finally, PEL cells are defined by the over-expression of genes involved in inflammation, cell adhesion and invasion, which is believed to contribute to their presentation in the body cavities [2]. Interestingly, in the present study, we observed significant upregulation of genes belonging to all the above categories upon K13 expression. Thus, K13 may contribute to the unique gene expression profile and presentation in body cavities that are characteristic of KSHV-associated PEL.

## Supporting Information

Table S1 **List of primers used for qRT-PCR (mRNA expression).**
(DOC)

Table S2 **Summary of differentially regulated gene clusters in 4OHT-treated K13-ER^TAM^-transduced BCBL1 cells.**
(DOC)

Table S3 **List of common differentially regulated genes in HUVECs and BCBL1 cells.**
(DOC)

Table S4 **List of genes upregulated >20 fold in HUVECs dataset.**
(DOC)

Table S5 **Summary of representative genes corresponding to pathways identified by GSEA analysis and validated in qRT-PCR.**
(DOCX)
