# Supplementary material for: A Computational Profiling of Changes in Gene Expression and Transcription Factors Induced by vFLIP K13 in Primary Effusion Lymphoma
Source: PLoS One. 2012 May 18;7(5):e37498. doi: 10.1371/journal.pone.0037498 (PMC3356309; doi:10.1371/journal.pone.0037498)
Supplement: Table S1 — List of primers used for qRT-PCR (mRNA expression). (DOC) [file pone.0037498.s001.doc]

| *Table S1. List of primers used for qRT-PCR (mRNA expression).* | | |
| --- | --- | --- |
| *S.No.* | *Primer name* | *Primer sequence* |
| 1 | VCAM1 Forward | GGGACCACATCTACGCTGACA |
| 2 | VCAM1 Reverse | CCTGTCTGCATCCTCCAGAAA |
| 3 | BIRC3 Forward | ACTTGAACAGCTGCTATCCACATC |
| 4 | BIRC3 Reverse | GTTGCTAGGATTTTTCTCTGAACTGTC |
| 5 | CCL5 Forward | CCCAGCAGTCGTCTTTGTCA |
| 6 | CCL5 Reverse | TCCCGAACCCATTTCTTCTCT |
| 7 | IL9 Forward | CTCTGTTTGGGCATTCCCTCT |
| 8 | IL9 Reverse | GGGTATCTTGTTTGCATGGTGG |
| 9 | IFNG Forward | TTGGCTTTTCAGCTCTGCAT |
| 10 | IFNG Reverse | CCGCTACATCTGAATGACCTG |
| 11 | IL15 Forward | CAGTGCAGGGCTTCCTAAAAC |
| 12 | IL15 Reverse | TGGGGTGAACATCACTTTCCG |
| 13 | NFKB1A Forward | TTACCCTCACCTTTTACTTCACATC |
| 14 | NFKB1A Reverse | AATGCAAGAGAGACCAGAGAAAGTA |
| 15 | NFKB1 Forward | GAAGCACGAATGACAGAGGC |
| 16 | NFKB1 Reverse | GCTTGGCGGATTAGCTCTTTT |
| 17 | TRADD Forward | GCTGTTTGAGTTGCATCCTAGC |
| 18 | TRADD Reverse | CCGCACTTCAGATTTCGCA |
| 19 | TNFRSF25 Forward | CTCACAAGCCCCTGGTTACTG |
| 20 | TNFSRF25 Reverse | GTTACCCACCAACTGGACGG |
| 21 | GAS2 Forward | CCTCCTGGTTTGATAAAGCTGG |
| 22 | GAS2 Reverse | CCCACTCGGTATCTTCCTTGG |
| 23 | TNFRSF1B Forward | CCGTGTGTGACTCCTGTGAG |
| 24 | TNFRSF1B Reverse | CAGATGCGGTTCTGTTCCC |
| 25 | LMNB2 Forward | TGACCAGAACGACAAGGCG |
| 26 | LMNB2 Reverse | CCGAATGCGATCTTCAGCG |
| 27 | BID Forward | CTCCGTGATGTCTTTCACACAA |
| 28 | BID Reverse | CGAGCTTTAGCCAGTCACACTT |
| 29 | HLADQA1 Forward | GGCTGTGGCAAAACACAACT |
| 30 | HLADQA1 Reverse | GTGTCACGGGAGACTTGGAA |
| 31 | CIITA Forward | CCTGGAGCTTCTTAACAGCGA |
| 32 | CIITA Reverse | TGTGTCGGGTTCTGAGTAGAG |
| 33 | CD74 Forward | CTCGTAGCTGAACAGCTGGACTAC |
| 34 | CD74 Reverse | TTCTTAAGGTGTCTCAGGTTCTCC |
| 35 | HLADMB Forward | AGCAGAGCATGATCACATTCCT |
| 36 | HLADMB Reverse | AACAGACAGGTGCTTTCCACAT |
| 37 | HLADBQB1 Forward | AGACTCTCCCGAGGATTTCGT |
| 38 | HLADBQB1 Reverse | GAAGTAGCACATGCCCTTAAACT |
| 39 | CTSS Forward | ATAACAAGGGCATCGACTCAGA |
| 40 | CTSS Reverse | TACTTTGAACATGTGGCAGCAC |
| 41 | SELE Forward | AGCCTAAACCTTTGGGTGAAAA |
| 42 | SELE Reverse | GTGTTGTAAGACCAGGCTCCAC |
| 43 | ALCAM Forward | TCCTGCCGTCTGCTCTTCT |
| 44 | ALCAM Reverse | TTCTGAGGTACGTCAAGTCGG |
| 45 | IL6 Forward | GGTACATCCTCGACGGCATCT |
| 46 | IL6 Reverse | GTGCCTCTTTGCTGCTTTCAC |
| 47 | LTbR Forward | GGAGACGACGAAGGAACAGG |
| 48 | LTbR Reverse | GTAGAGGTAATAGAGGCCGTCC |
| 49 | CXCL10 Forward | GAGCCTACAGCAGAGGAACC |
| 50 | CXCL10 Reverse | GAGTCAGAAAGATAAGGCAGC |
| 51 | GAPDH Forward | TACTAGCGGTTTTACGGGCG |
| 52 | GAPDH Reverse | TCGAACAGGAGGAGCAGAGAGCGA |
| 53 | B2M Forward | ACTGAATTCACCCCCACTGA |
| 54 | B2M Reverse | CCTCCATGATGCTGCTTACA |
| 55 | GNB2L Forward | GAGTGTGGCCTTCTCCTCTG |
| 56 | GNB2L Reverse | GCTTGCAGTTAGCCAGGTTC |
| 57 | 18S RNA Forward | GATATGCTCATGTGGTGTTG |
| 58 | 18S RNA Reverse | AATCTTCTTCAGTCGCTCCA |
