# Supplementary material for: A Computational Profiling of Changes in Gene Expression and Transcription Factors Induced by vFLIP K13 in Primary Effusion Lymphoma
Source: PLoS One. 2012 May 18;7(5):e37498. doi: 10.1371/journal.pone.0037498 (PMC3356309; doi:10.1371/journal.pone.0037498)
Supplement: Table S2 — Summary of differentially regulated gene clusters in 4OHT-treated K13-ERTAM-transduced BCBL1 cells. (DOC) [file pone.0037498.s002.doc]

| *Table S2. Summary of differentially regulated gene clusters in 4OHT-treated K13-ER^TAM^-transduced BCBL1 cells.* | | | | | | |
| --- | --- | --- | --- | --- | --- | --- |
| *S.No.* | *Entrez Gene* | *Gene Symbol* | *Gene Title* | *RefSeq Transcript ID* | *Fold change* | *Regu-*  *lation* |
| 1. | 10537 | UBD | ubiquitin D | NM_001470 | 26.07 | up |
| 2. | 5328 | PLAU | plasminogen activator, urokinase | NM_001145031 | 19.66 | up |
| 3. | 5645 | PRSS2 | protease, serine, 2 (trypsin 2) | NM_002770 | 14.84 | up |
| 4. | 330 | BIRC3 | baculoviral IAP repeat-containing 3 | NM_001165 | 13.51 | up |
| 5. | 5644 | PRSS1 | protease, serine, 1 (trypsin 1) | NM_002769 | 13.24 | up |
| 6. | 4050 | LTB | lymphotoxin beta (TNF superfamily, member 3) | NM_002341 | 12.98 | up |
| 7. | 7128 | TNFAIP3 | tumor necrosis factor, alpha-induced protein 3 | NM_006290 | 12.85 | up |
| 8. | 154754 | PRSS1 /// TRY6 | protease, serine, 1 (trypsin 1) | NM_002769 | 12.56 | up |
| 9. | 7262 | PHLDA2 | pleckstrin homology-like domain, family A, member 2 | NM_003311 | 10.51 | up |
| 10. | 10148 | EBI3 | Epstein-Barr virus induced 3 | NM_005755 | 9.90 | up |
| 11. | 4914 | NTRK1 | neurotrophic tyrosine kinase, receptor, type 1 | NM_001007792 | 9.51 | up |
| 12. | 6352 | CCL5 | chemokine (C-C motif) ligand 5 | NM_002985 | 9.42 | up |
| 13. | 4792 | NFKBIA | nuclear factor of kappa light polypeptide gene enhancer in B-cells inhibitor, alpha | NM_020529 | 8.33 | up |
| 14. | 2015 | EMR1 | egf-like module containing, mucin-like, hormone receptor-like 1 | NM_001974 | 8.10 | up |
| 15. | 355 | FAS | Fas (TNF receptor superfamily, member 6) | NM_000043 | 7.94 | up |
| 16. | 972 | CD74 | CD74 molecule, major histocompatibility complex, class II invariant chain | NM_001025158 | 7.02 | up |
| 17. | 3117 | HLA-DQA1 | major histocompatibility complex, class II, DQ alpha 1 | NM_002122 | 6.73 | up |
| 18. | 259307 | IL4I1 | interleukin 4 induced 1 | NM_152899 | 6.58 | up |
| 19. | 51700 | CYB5R2 | cytochrome b5 reductase 2 | NM_016229 | 6.44 | up |
| 20. | 1543 | CYP1A1 | cytochrome P450, family 1, subfamily A, polypeptide 1 | NM_000499 | 6.01 | up |
| 21. | 25907 | TMEM158 | transmembrane protein 158 | NM_015444 | 5.81 | up |
| 22. | 7273 | TTN | Titin | NM_003319 | 5.21 | up |
| 23. | 84033 | OBSCN | obscurin, cytoskeletal calmodulin and titin-interacting RhoGEF | NM_001098623 | 5.17 | up |
| 24. | 7412 | VCAM1 | vascular cell adhesion molecule 1 | NM_001078 | 5.07 | up |
| 25. | 9294 | S1PR2 | sphingosine-1-phosphate receptor 2 | NM_004230 | 5.03 | up |
| 26. | 5971 | RELB | v-rel reticuloendotheliosis viral oncogene homolog B | NM_006509 | 4.97 | up |
| 27. | 5996 | RGS1 | regulator of G-protein signaling 1 | NM_002922 | 4.97 | up |
| 28. | 9308 | CD83 | CD83 molecule | NM_001040280 | 4.94 | up |
| 29. | 3383 | ICAM1 | intercellular adhesion molecule 1 | NM_000201 | 4.93 | up |
| 30. | 25801 | GCA | grancalcin, EF-hand calcium binding protein | NM_012198 | 4.92 | up |
| 31. | 9235 | IL32 | interleukin 32 | NM_001012631 | 4.81 | up |
| 32. | 8651 | SOCS1 | suppressor of cytokine signaling 1 | NM_003745 | 4.75 | up |
| 33. | 11226 | GALNT6 | UDP-N-acetyl-alpha-D-galactosamine:polypeptide N-acetylgalactosaminyltransferase 6 | NM_007210 | 4.70 | up |
| 34. | 1999 | ELF3 | E74-like factor 3 (ets domain transcription factor, epithelial-specific ) | NM_001114309 | 4.68 | up |
| 35. | 3109 | HLA-DMB | major histocompatibility complex, class II, DM beta | NM_002118 | 4.50 | up |
| 36. | 115019 | SLC26A9 | solute carrier family 26, member 9 | NM_001142600 | 4.49 | up |
| 37. | 3561 | IL2RG | interleukin 2 receptor, gamma (severe combined immunodeficiency) | NM_000206 | 4.48 | up |
| 38. | 4261 | CIITA | class II, major histocompatibility complex, transactivator | NM_000246 | 4.34 | up |
| 39. | 4791 | NFKB2 | nuclear factor of kappa light polypeptide gene enhancer in B-cells 2 (p49/p100) | NM_001077493 | 4.29 | up |
| 40. | 718 | C3 | complement component 3 | NM_000064 | 4.29 | up |
| 41. | 285025 | CCDC141 | coiled-coil domain containing 141 | NM_173648 | 4.29 | up |
| 42. | 100132999 | LOC100132999 | hypothetical protein LOC100132999 | XM_001722799 | 4.16 | up |
| 43. | 6892 | TAPBP | TAP binding protein (tapasin) | NM_003190 | 4.14 | up |
| 44. | 6236 | RRAD | Ras-related associated with diabetes | NM_001128850 | 4.12 | up |
| 45. | 27074 | LAMP3 | lysosomal-associated membrane protein 3 | NM_014398 | 3.99 | up |
| 46. | 2232 | FDXR | ferredoxin reductase | NM_004110 | 3.98 | up |
| 47. | 3119 | HLA-DQB1 | major histocompatibility complex, class II, DQ beta 1 | NM_002123 | 3.77 | up |
| 48. | 114294 | LACTB | lactamase, beta | NM_032857 | 3.70 | up |
| 49. | 64319 | FBRS | Fibrosin | NM_001105079 | 3.70 | up |
| 50. | 780 | DDR1 | discoidin domain receptor tyrosine kinase 1 | NM_001954 | 3.68 | up |
| 51. | 9972 | NUP153 | nucleoporin 153kDa | NM_005124 | 3.67 | up |
| 52. | 11074 | TRIM31 | tripartite motif-containing 31 | NM_007028 | 3.63 | up |
| 53. | 121214 | SDR9C7 | short chain dehydrogenase/reductase family 9C, member 7 | NM_148897 | 3.62 | up |
| 54. | 1326 | MAP3K8 | mitogen-activated protein kinase kinase kinase 8 | NM_005204 | 3.55 | up |
| 55. | 9047 | SH2D2A | SH2 domain protein 2A | NM_003975 | 3.55 | up |
| 56. | 3627 | CXCL10 | chemokine (C-X-C motif) ligand 10 | NM_001565 | 3.55 | up |
| 57. | 440279 | UNC13C | unc-13 homolog C (C. elegans) | NM_001080534 | 3.53 | up |
| 58. | 9429 | ABCG2 | ATP-binding cassette, sub-family G (WHITE) | NM_004827 | 3.53 | up |
| 59. | 222029 | DKFZp434L192 | hypothetical protein DKFZp434L192 | NR_026929 | 3.52 | up |
| 60. | 2669 | GEM | GTP binding protein overexpressed in skeletal muscle | NM_005261 | 3.49 | up |
| 61. | 8870 | IER3 | immediate early response 3 | NM_003897 | 3.48 | up |
| 62. | 22898 | DENND3 | DENN/MADD domain containing 3 | NM_014957 | 3.47 | up |
| 63. | 6376 | CX3CL1 | chemokine (C-X3-C motif) ligand 1 | NM_002996 | 3.44 | up |
| 64. | 26166 | RGS22 | regulator of G-protein signaling 22 | NM_015668 | 3.44 | up |
| 65. | 10865 | ARID5A | AT rich interactive domain 5A (MRF1-like) | NM_212481 | 3.35 | up |
| 66. | 3108 | HLA-DMA | major histocompatibility complex, class II, DM alpha | NM_006120 | 3.32 | up |
| 67. | 285966 | FAM115C | family with sequence similarity 115, member C | NM_001130025 | 3.25 | up |
| 68. | 3361 | HTR5A | 5-hydroxytryptamine (serotonin) receptor 5A | NM_024012 | 3.25 | up |
| 69. | 79668 | PARP8 | Poly (ADP-ribose) polymerase family, member 8 | NM_024615 | 3.25 | up |
| 70. | 124944 | C17orf49 | chromosome 17 open reading frame 49 | NM_001142798 | 3.24 | up |
| 71. | 9466 | IL27RA | interleukin 27 receptor, alpha | NM_004843 | 3.23 | up |
| 72. | 901 | CCNG2 | cyclin G2 | NM_004354 | 3.21 | up |
| 73. | 1956 | EGFR | epidermal growth factor receptor (erythroblastic leukemia viral (v-erb-b) oncogene homolog) | NM_005228 | 3.10 | up |
| 74. | 4794 | NFKBIE | nuclear factor of kappa light polypeptide gene enhancer in B-cells inhibitor, epsilon | NM_004556 | 3.08 | up |
| 75. | 3487 | IGFBP4 | insulin-like growth factor binding protein 4 | NM_001552 | 3.06 | up |
| 76. | 285628 | LOC285628 | hypothetical protein LOC285628 |  | 3.06 | up |
| 77. | 1281 | COL3A1 | collagen, type III, alpha 1 | NM_000090 | 3.03 | up |
| 78. | 154761 | LOC154761 | hypothetical LOC154761 | NR_015421 | 3.01 | up |
| 79. | 149233 | IL23R | interleukin 23 receptor | NM_144701 | 2.96 | up |
| 80. | 10809 | STARD10 | StAR-related lipid transfer (START) domain containing 10 | NM_006645 | 2.93 | up |
| 81. | 1277 | COL1A1 | collagen, type I, alpha 1 | NM_000088 | 2.92 | up |
| 82. | 9641 | IKBKE | inhibitor of kappa light polypeptide gene enhancer in B-cells, kinase epsilon | NM_014002 | 2.90 | up |
| 83. | 64332 | NFKBIZ | nuclear factor of kappa light polypeptide gene enhancer in B-cells inhibitor, zeta | NM_001005474 | 2.87 | up |
| 84. | 4814 | NINJ1 | ninjurin 1 | NM_004148 | 2.85 | up |
| 85. | 3663 | IRF5 | interferon regulatory factor 5 | NM_001098627 | 2.83 | up |
| 86. | 26330 | GAPDHS | glyceraldehyde-3-phosphate dehydrogenase, spermatogenic | NM_014364 | 2.82 | up |
| 87. | 5734 | PTGER4 | prostaglandin E receptor 4 (subtype EP4) | NM_000958 | 2.81 | up |
| 88. | 3726 | JUNB | jun B proto-oncogene | NM_002229 | 2.79 | up |
| 89. | 3604 | TNFRSF9 | tumor necrosis factor receptor superfamily, member 9 | NM_001561 | 2.78 | up |
| 90. | 340090 | LOC340090 | hypothetical protein LOC340090 |  | 2.77 | up |
| 91. | 151887 | CCDC80 | coiled-coil domain containing 80 | NM_199511 | 2.76 | up |
| 92. | 4902 | NRTN | Neurturin | NM_004558 | 2.74 | up |
| 93. | 123920 | CMTM3 | CKLF-like MARVEL transmembrane domain containing 3 | NM_001048251 | 2.73 | up |
| 94. | 85360 | SYDE1 | synapse defective 1, Rho GTPase, homolog 1 (C. elegans) | NM_033025 | 2.73 | up |
| 95. | 64135 | IFIH1 | interferon induced with helicase C domain 1 | NM_022168 | 2.72 | up |
| 96. | 6615 | SNAI1 | snail homolog 1 (Drosophila) | NM_005985 | 2.71 | up |
| 97. | 126282 | TNFAIP8L1 | tumor necrosis factor, alpha-induced protein 8-like 1 | NM_152362 | 2.70 | up |
| 98. | 1466 | CSRP2 | cysteine and glycine-rich protein 2 | NM_001321 | 2.69 | up |
| 99. | 85479 | DNAJC5B | DnaJ (Hsp40) homolog, subfamily C, member 5 beta | NM_033105 | 2.67 | up |
| 100. | 4544 | MTNR1B | melatonin receptor 1B | NM_005959 | 2.67 | up |
| 101. | 4487 | MSX1 | msh homeobox 1 | NM_002448 | 2.66 | up |
| 102. | 7480 | WNT10B | wingless-type MMTV integration site family, member 10B | NM_003394 | 2.65 | up |
| 103. | 57151 | LYZL6 | lysozyme-like 6 | NM_020426 | 2.65 | up |
| 104. | 196446 | C12orf28 | chromosome 12 open reading frame 28 | XM_001716650 | 2.64 | up |
| 105. | 3113 | HLA-DPA1 | major histocompatibility complex, class II, DP alpha 1 | NM_033554 | 2.62 | up |
| 106. | 84649 | DGAT2 | diacylglycerol O-acyltransferase homolog 2 (mouse) | NM_032564 | 2.61 | up |
| 107. | 3122 | HLA-DRA | major histocompatibility complex, class II, DR alpha | NM_019111 | 2.60 | up |
| 108. | 57402 | S100A14 | S100 calcium binding protein A14 | NM_020672 | 2.56 | up |
| 109. | 8418 | CMAH | cytidine monophosphate-N-acetylneuraminic acid hydroxylase pseudogene | NR_002174 | 2.56 | up |
| 110. | 83637 | ZMIZ2 | zinc finger, MIZ-type containing 2 | NM_031449 | 2.55 | up |
| 111. | 4046 | LSP1 | lymphocyte-specific protein 1 | NM_001013253 | 2.55 | up |
| 112. | 3134 | HLA-F | major histocompatibility complex, class I, F | NM_001098478 | 2.55 | up |
| 113. | 9914 | ATP2C2 | ATPase, Ca++ transporting, type 2C, member 2 | NM_014861 | 2.55 | up |
| 114. | 728492 | SERF1A | small EDRK-rich factor 1A (telomeric) | NM_021967 | 2.51 | up |
| 115. | 7067 | NR1D1 | nuclear receptor subfamily 1, group D, member 1 | NM_003250 | 2.48 | up |
| 116. | 260293 | CYP4X1 | cytochrome P450, family 4, subfamily X, polypeptide 1 | NM_178033 | 2.48 | up |
| 117. | 2907 | GRINA | glutamate receptor, ionotropic | NM_000837 | 2.46 | up |
| 118. | 814 | CAMK4 | calcium/calmodulin-dependent protein kinase IV | NM_001744 | 2.46 | up |
| 119. | 3111 | HLA-DOA | major histocompatibility complex, class II, DO alpha | NM_002119 | 2.46 | up |
| 120. | 8876 | VNN1 | vanin 1 | NM_004666 | 2.45 | up |
| 121. | 3115 | HLA-DPB1 | major histocompatibility complex, class II, DP beta 1 | NM_002121 | 2.45 | up |
| 122. | 29923 | C7orf68 | chromosome 7 open reading frame 68 | NM_001098786 | 2.45 | up |
| 123. | 7046 | TGFBR1 | transforming growth factor, beta receptor 1 | NM_001130916 | 2.44 | up |
| 124. | 9143 | SYNGR3 | synaptogyrin 3 | NM_004209 | 2.44 | up |
| 125. | 3855 | KRT7 | keratin 7 | NM_005556 | 2.43 | up |
| 126. | 353322 | ANKRD37 | ankyrin repeat domain 37 | NM_181726 | 2.42 | up |
| 127. | 100133484 | HLA-DRB1 | major histocompatibility complex, class II, DR beta 1 | NM_002124 | 2.41 | up |
| 128. | 389362 | PSMG4 | proteasome (prosome, macropain) assembly chaperone 4 | NM_001128591 | 2.40 | up |
| 129. | 159686 | CCDC147 | coiled-coil domain containing 147 | NM_001008723 | 2.40 | up |
| 130. | 9582 | APOBEC3B | apolipoprotein B mRNA editing enzyme, catalytic polypeptide-like 3B | NM_004900 | 2.40 | up |
| 131. | 117245 | HRASLS5 | HRAS-like suppressor family, member 5 | NM_001146728 | 2.39 | up |
| 132. | 3535 | IGL@ | Immunoglobulin lambda locus |  | 2.38 | up |
| 133. | 634 | CEACAM1 | carcinoembryonic antigen-related cell adhesion molecule 1 (biliary glycoprotein) | NM_001024912 | 2.38 | up |
| 134. | 643866 | CBLN3 | cerebellin 3 precursor | NM_001039771 | 2.37 | up |
| 135. | 27319 | BHLHE22 | basic helix-loop-helix family, member e22 | NM_152414 | 2.36 | up |
| 136. | 84162 | KIAA1109 | KIAA1109 | NM_015312 | 2.36 | up |
| 137. | 221016 | CCDC7 | coiled-coil domain containing 7 | NM_001026383 | 2.36 | up |
| 138. | 26493 | OR8B8 | olfactory receptor, family 8, subfamily B, member 8 | NM_012378 | 2.35 | up |
| 139. | 113791 | PIK3IP1 | phosphoinositide-3-kinase interacting protein 1 | NM_001135911 | 2.34 | up |
| 140. | 3096 | HIVEP1 | human immunodeficiency virus type I enhancer binding protein 1 | NM_002114 | 2.34 | up |
| 141. | 440888 | LOC440888 | ARP3 actin-related protein 3 homolog B pseudogene |  | 2.34 | up |
| 142. | 57217 | TTC7A | tetratricopeptide repeat domain 7A | NM_020458 | 2.32 | up |
| 143. | 1236 | CCR7 | chemokine (C-C motif) receptor 7 | NM_001838 | 2.31 | up |
| 144. | 84984 | C3orf34 | chromosome 3 open reading frame 34 | NM_032898 | 2.30 | up |
| 145. | 51305 | KCNK9 | Potassium channel, subfamily K, member 9 | NM_016601 | 2.30 | up |
| 146. | 282980 | LOC282980 | hypothetical protein LOC282980 |  | 2.29 | up |
| 147. | 22822 | PHLDA1 | pleckstrin homology-like domain, family A, member 1 | NM_007350 | 2.29 | up |
| 148. | 9536 | PTGES | prostaglandin E synthase | NM_004878 | 2.29 | up |
| 149. | 84868 | HAVCR2 | hepatitis A virus cellular receptor 2 | NM_032782 | 2.28 | up |
| 150. | 117584 | RFFL | ring finger and FYVE-like domain containing 1 | NM_001017368 | 2.28 | up |
| 151. | 51523 | CXXC5 | CXXC finger 5 | NM_016463 | 2.28 | up |
| 152. | 80326 | WNT10A | wingless-type MMTV integration site family, member 10A | NM_025216 | 2.27 | up |
| 153. | 4583 | MUC2 | mucin 2, oligomeric mucus/gel-forming | NM_002457 | 2.27 | up |
| 154. | 200298 | C22orf37 | chromosome 22 open reading frame 37 | XR_040827 | 2.26 | up |
| 155. | 8875 | VNN2 | vanin 2 | NM_004665 | 2.26 | up |
| 156. | 8293 | SERF1A | small EDRK-rich factor 1A (telomeric) | NM_021967 | 2.25 | up |
| 157. | 84102 | SLC41A2 | solute carrier family 41, member 2 | NM_032148 | 2.24 | up |
| 158. | 10318 | TNIP1 | TNFAIP3 interacting protein 1 | NM_006058 | 2.24 | up |
| 159. | 285965 | LOC285965 | hypothetical protein LOC285965 |  | 2.24 | up |
| 160. | 59 | ACTA2 | actin, alpha 2, smooth muscle, aorta | NM_001141945 | 2.24 | up |
| 161. | 130340 | AP1S3 | adaptor-related protein complex 1, sigma 3 subunit | NM_001039569 | 2.23 | up |
| 162. | 1193 | CLIC2 | chloride intracellular channel 2 | NM_001289 | 2.23 | up |
| 163. | 58506 | SCAF1 | SR-related CTD-associated factor 1 | NM_021228 | 2.22 | up |
| 164. | 1520 | CTSS | cathepsin S | NM_004079 | 2.22 | up |
| 165. | 671 | BPI | bactericidal/permeability-increasing protein | NM_001725 | 2.22 | up |
| 166. | 1601 | DAB2 | disabled homolog 2, mitogen-responsive phosphoprotein (Drosophila) | NM_001343 | 2.22 | up |
| 167. | 284751 | LOC284751 | hypothetical LOC284751 | NM_001025463 | 2.21 | up |
| 168. | 23193 | GANAB | glucosidase, alpha; neutral AB | NM_198334 | 2.21 | up |
| 169. | 116496 | FAM129A | family with sequence similarity 129, member A | NM_052966 | 2.21 | up |
| 170. | 5089 | PBX2 | pre-B-cell leukemia homeobox 2 | NM_002586 | 2.20 | up |
| 171. | 123775 | C16orf46 | chromosome 16 open reading frame 46 | NM_001100873 | 2.20 | up |
| 172. | 55615 | PRR5 | proline rich 5 (renal) | NM_001017528 | 2.20 | up |
| 173. | 7475 | WNT6 | wingless-type MMTV integration site family, member 6 | NM_006522 | 2.20 | up |
| 174. | 25797 | QPCT | glutaminyl-peptide cyclotransferase | NM_012413 | 2.19 | up |
| 175. | 10741 | RBBP9 | retinoblastoma binding protein 9 | NM_006606 | 2.19 | up |
| 176. | 3566 | IL4R | interleukin 4 receptor | NM_000418 | 2.18 | up |
| 177. | 197135 | PATL2 | protein associated with topoisomerase II homolog 2 (yeast) | NM_001145112 | 2.18 | up |
| 178. | 2915 | GRM5 | glutamate receptor, metabotropic 5 | NM_000842 | 2.18 | up |
| 179. | 57829 | ZP4 | zona pellucida glycoprotein 4 | NM_021186 | 2.18 | up |
| 180. | 9120 | SLC16A6 | solute carrier family 16, member 6 | NM_004694 | 2.17 | up |
| 181. | 5600 | MAPK11 | mitogen-activated protein kinase 11 | NM_002751 | 2.17 | up |
| 182. | 91748 | C14orf43 | chromosome 14 open reading frame 43 | NM_001043318 | 2.17 | up |
| 183. | 55176 | SEC61A2 | Sec61 alpha 2 subunit (S. cerevisiae) | NM_001142627 | 2.17 | up |
| 184. | 121268 | RHEBL1 | Ras homolog enriched in brain like 1 | NM_144593 | 2.16 | up |
| 185. | 5366 | PMAIP1 | phorbol-12-myristate-13-acetate-induced protein 1 | NM_021127 | 2.16 | up |
| 186. | 4703 | NEB | Nebulin | NM_004543 | 2.16 | up |
| 187. | 619426 | C8orf60 | chromosome 8 open reading frame 60 |  | 2.16 | up |
| 188. | 54714 | CNGB3 | cyclic nucleotide gated channel beta 3 | NM_019098 | 2.16 | up |
| 189. | 9923 | ZBTB40 | zinc finger and BTB domain containing 40 | NM_001083621 | 2.16 | up |
| 190. | 149708 | WFDC5 | WAP four-disulfide core domain 5 | NM_145652 | 2.15 | up |
| 191. | 27245 | AHDC1 | AT hook, DNA binding motif, containing 1 | NM_001029882 | 2.15 | up |
| 192. | 2256 | FGF11 | fibroblast growth factor 11 | NM_004112 | 2.14 | up |
| 193. | 56938 | ARNTL2 | aryl hydrocarbon receptor nuclear translocator-like 2 | NM_020183 | 2.14 | up |
| 194. | 387895 | LOC387895 | hypothetical gene supported by BC040060 | XM_373553 | 2.13 | up |
| 195. | 100132074 | FOXO6 | forkhead box protein O6 |  | 2.13 | up |
| 196. | 146754 | DNAH2 | dynein, axonemal, heavy chain 2 | NM_020877 | 2.13 | up |
| 197. | 201191 | SAMD14 | sterile alpha motif domain containing 14 | NM_174920 | 2.13 | up |
| 198. | 100133744 | EFCAB3 | EF-hand calcium binding domain 3 /// similar to hypoxia-inducible protein 2 | NM_001144933 | 2.13 | up |
| 199. | 10071 | MUC12 | mucin 12, cell surface associated | XM_001714400 | 2.13 | up |
| 200. | 10220 | GDF11 | growth differentiation factor 11 | NM_005811 | 2.12 | up |
| 201. | 5646 | PRSS3 | protease, serine, 3 | NM_002771 | 2.12 | up |
| 202. | 5900 | RALGDS | ral guanine nucleotide dissociation stimulator | NM_001042368 | 2.12 | up |
| 203. | 999 | CDH1 | cadherin 1, type 1, E-cadherin (epithelial) | NM_004360 | 2.11 | up |
| 204. | 3219 | HOXB9 | homeobox B9 | NM_024017 | 2.11 | up |
| 205. | 3128 | HLA-DRB6 | major histocompatibility complex, class II, DR beta 6 (pseudogene) | NR_001298 | 2.11 | up |
| 206. | 3123 | HLA-DRB1 | major histocompatibility complex, class II, DR beta 1 | NM_002124 | 2.10 | up |
| 207. | 121260 | SLC15A4 | solute carrier family 15, member 4 | NM_145648 | 2.10 | up |
| 208. | 23732 | C9orf4 | chromosome 9 open reading frame 4 | NM_014334 | 2.10 | up |
| 209. | 953 | ENTPD1 | ectonucleoside triphosphate diphosphohydrolase 1 | NM_001098175 | 2.10 | up |
| 210. | 2628 | GATM | glycine amidinotransferase (L-arginine:glycine amidinotransferase) | NM_001482 | 2.09 | up |
| 211. | 115290 | FBXO17 | F-box protein 17 | NM_024907 | 2.09 | up |
| 212. | 83541 | FAM110A | family with sequence similarity 110, member A | NM_001042353 | 2.09 | up |
| 213. | 5265 | SERPINA1 | serpin peptidase inhibitor, clade A (alpha-1 antiproteinase, antitrypsin), member 1 | NM_000295 | 2.09 | up |
| 214. | 140700 | SAMD10 | sterile alpha motif domain containing 10 | NM_080621 | 2.08 | up |
| 215. | 139170 | WDR40B | WD repeat domain 40B | NM_178470 | 2.08 | up |
| 216. | 100129899 | LOC100129899 | hypothetical protein LOC100129899 | XM_001714827 | 2.08 | up |
| 217. | 117579 | RLN3 | relaxin 3 | NM_080864 | 2.08 | up |
| 218. | 30814 | PLA2G2E | phospholipase A2, group IIE | NM_014589 | 2.07 | up |
| 219. | 90196 | SYS1 | SYS1 Golgi-localized integral membrane protein homolog (S. cerevisiae) | NM_033542 | 2.06 | up |
| 220. | 222537 | HS3ST5 | heparan sulfate (glucosamine) 3-O-sulfotransferase 5 | NM_153612 | 2.06 | up |
| 221. | 80319 | CXXC4 | CXXC finger 4 | NM_025212 | 2.06 | up |
| 222. | 10626 | TRIM16 | tripartite motif-containing 16 | NM_006470 | 2.06 | up |
| 223. | 9242 | MSC | musculin (activated B-cell factor-1) | NM_005098 | 2.05 | up |
| 224. | 10272 | FSTL3 | follistatin-like 3 (secreted glycoprotein) | NM_005860 | 2.05 | up |
| 225. | 164781 | WDR69 | WD repeat domain 69 | NM_178821 | 2.05 | up |
| 226. | 4258 | MGST2 | microsomal glutathione S-transferase 2 | NM_002413 | 2.05 | up |
| 227. | 79623 | GALNT14 | UDP-N-acetyl-alpha-D-galactosamine:polypeptide N-acetylgalactosaminyltransferase 14 | NM_024572 | 2.05 | up |
| 228. | 64231 | MS4A6A | membrane-spanning 4-domains, subfamily A, member 6A | NM_022349 | 2.05 | up |
| 229. | 100129286 | LOC100129286 | Hypothetical protein LOC100129286 | XM_001724487 | 2.05 | up |
| 230. | 84335 | AKT1S1 | AKT1 substrate 1 (proline-rich) | NM_001098632 | 2.05 | up |
| 231. | 9144 | SYNGR2 | synaptogyrin 2 | NM_004710 | 2.05 | up |
| 232. | 64375 | IKZF4 | IKAROS family zinc finger 4 (Eos) | NM_022465 | 2.04 | up |
| 233. | 4311 | MME | membrane metallo-endopeptidase | NM_000902 | 2.04 | up |
| 234. | 414236 | C10orf55 | chromosome 10 open reading frame 55 | NM_001001791 | 2.04 | up |
| 235. | 286128 | ZFP41 | zinc finger protein 41 homolog (mouse) | NM_173832 | 2.04 | up |
| 236. | 9546 | APBA3 | amyloid beta (A4) precursor protein-binding, family A, member 3 | NM_004886 | 2.04 | up |
| 237. | 3437 | IFIT3 | interferon-induced protein with tetratricopeptide repeats 3 | NM_001031683 | 2.04 | up |
| 238. | 79156 | PLEKHF1 | pleckstrin homology domain containing, family F (with FYVE domain) member 1 | NM_024310 | 2.03 | up |
| 239. | 50717 | WDR42A | WD repeat domain 42A | NM_015726 | 2.03 | up |
| 240. | 57007 | CXCR7 | chemokine (C-X-C motif) receptor 7 | NM_020311 | 2.03 | up |
| 241. | 51278 | IER5 | immediate early response 5 | NM_016545 | 2.03 | up |
| 242. | 602 | BCL3 | B-cell CLL/lymphoma 3 | NM_005178 | 2.03 | up |
| 243. | 149473 | CCDC24 | coiled-coil domain containing 24 | NM_152499 | 2.03 | up |
| 244. | 284260 | LOC284260 | hypothetical gene supported by BC011527; BC021928; BC011527 | XM_001720683 | 2.03 | up |
| 245. | 92346 | C1orf105 | chromosome 1 open reading frame 105 | NM_139240 | 2.02 | up |
| 246. | 8492 | PRSS12 | protease, serine, 12 (neurotrypsin, motopsin) | NM_003619 | 2.02 | up |
| 247. | 65264 | UBE2Z | ubiquitin-conjugating enzyme E2Z | NM_023079 | 2.02 | up |
| 248. | 100133005 | RASA4 | RAS p21 protein activator 4 pseudogene /// RAS p21 protein activator 4 | NM_001079877 | 2.01 | up |
| 249. | 970 | CD70 | CD70 molecule | NM_001252 | 2.01 | up |
| 250. | 27429 | HTRA2 | HtrA serine peptidase 2 | NM_013247 | 2.01 | up |
| 251. | 4745 | NELL1 | NEL-like 1 (chicken) | NM_006157 | 2.01 | up |
| 252. | 340152 | PPIL4 | peptidylprolyl isomerase (cyclophilin)-like 4 | NM_139126 | 2.01 | up |
| 253. | 727933 | LOC727933 | hypothetical LOC727933 | XM_001128972 | 2.01 | up |
| 254. | 8189 | SYMPK | Symplekin | NM_004819 | 2.01 | up |
| 255. | 79365 | BHLHE41 | basic helix-loop-helix family, member e41 | NM_030762 | 2.00 | up |
| 256. | 11124 | FAF1 | Fas (TNFRSF6) associated factor 1 | NM_007051 | 3.31 | down |
| 257. | 55916 | NXT2 | nuclear transport factor 2-like export factor 2 | NM_018698 | 2.74 | down |
| 258. | 51616 | TAF9B | TAF9B RNA polymerase II, TATA box binding protein (TBP)-associated factor, 31kDa | NM_015975 | 2.71 | down |
| 259. | 2162 | F13A1 | coagulation factor XIII, A1 polypeptide | NM_000129 | 2.63 | down |
| 260. | 3753 | KCNE1 | potassium voltage-gated channel, Isk-related family, member 1 | NM_000219 | 2.53 | down |
| 261. | 55737 | VPS35 | vacuolar protein sorting 35 homolog (S. cerevisiae) | NM_018206 | 2.45 | down |
| 262. | 51474 | LIMA1 | LIM domain and actin binding 1 | NM_001113546 | 2.43 | down |
| 263. | 135932 | TMEM139 | transmembrane protein 139 | NM_153345 | 2.38 | down |
| 264. | 2844 | GPR21 | G protein-coupled receptor 21 | NM_005294 | 2.34 | down |
| 265. | 930 | CD19 | CD19 molecule | NM_001770 | 2.32 | down |
| 266. | 6711 | SPTBN1 | spectrin, beta, non-erythrocytic 1 | NM_003128 | 2.30 | down |
| 267. | 2744 | GLS | Glutaminase | NM_014905 | 2.29 | down |
| 268. | 3890 | KRT84 | keratin 84 | NM_033045 | 2.23 | down |
| 269. | 54704 | PPM2C | protein phosphatase 2C, magnesium-dependent, catalytic subunit | NM_018444 | 2.23 | down |
| 270. | 1131 | CHRM3 | cholinergic receptor, muscarinic 3 | NM_000740 | 2.22 | down |
| 271. | 129807 | NEU4 | sialidase 4 | NM_080741 | 2.21 | down |
| 272. | 135114 | HINT3 | histidine triad nucleotide binding protein 3 | NM_138571 | 2.19 | down |
| 273. | 5567 | PRKACB | protein kinase, cAMP-dependent, catalytic, beta | NM_002731 | 2.13 | down |
| 274. | 168374 | ZNF92 | zinc finger protein 92 | NM_007139 | 2.13 | down |
| 275. | 117608 | ZNF354B | zinc finger protein 354B | NM_058230 | 2.13 | down |
| 276. | 5579 | PRKCB | protein kinase C, beta | NM_002738 | 2.13 | down |
| 277. | 3308 | HSPA4 | heat shock 70kDa protein 4 | NM_002154 | 2.13 | down |
| 278. | 11156 | PTP4A3 | protein tyrosine phosphatase type IVA, member 3 | NM_007079 | 2.11 | down |
| 279. | 9949 | AMMECR1 | Alport syndrome, mental retardation | NM_001025580 | 2.11 | down |
| 280. | 7434 | VIPR2 | vasoactive intestinal peptide receptor 2 | NM_003382 | 2.01 | down |
| 281. | 8994 | LIMD1 | LIM domains containing 1 | NM_014240 | 2.08 | down |
| 282. | 6638 | SNRPN | small nuclear ribonucleoprotein polypeptide N | NM_003097 | 2.04 | down |
| 283. | 4144 | MAT2A | methionine adenosyltransferase II, alpha | NM_005911 | 2.04 | down |
| 284. | 3467 | IFNW1 | interferon, omega 1 | NM_002177 | 2.03 | down |
| 285. | 200879 | LIPH | lipase, member H | NM_139248 | 2.03 | down |
| 286. | 1592 | CYP26A1 | cytochrome P450, family 26, subfamily A, polypeptide 1 | NM_000783 | 2.03 | down |
| 287. | 3931 | LCAT | Lecithin-cholesterol acyltransferase | NM_000229 | 2.03 | down |
| 288. | 148789 | B3GALNT2 | beta-1,3-N-acetylgalactosaminyltransferase 2 | NM_152490 | 2.01 | down |
| 289. | 4033 | LRMP | lymphoid-restricted membrane protein | NM_006152 | 2.01 | down |
| 290. | 64853 | AIDA | axin interactor, dorsalization associated | NM_022831 | 2.00 | down |
| 291. | 9782 | MATR3 | matrin 3 | NM_018834 | 2.00 | down |
