# Supplementary material for: A Computational Profiling of Changes in Gene Expression and Transcription Factors Induced by vFLIP K13 in Primary Effusion Lymphoma
Source: PLoS One. 2012 May 18;7(5):e37498. doi: 10.1371/journal.pone.0037498 (PMC3356309; doi:10.1371/journal.pone.0037498)
Supplement: Table S3 — List of common differentially regulated genes in HUVECs and BCBL1 cells. (DOC) [file pone.0037498.s003.doc]

| *Table S3. List of common differentially regulated genes in HUVECs and BCBL1 cells.* | | | | | | | | |
| --- | --- | --- | --- | --- | --- | --- | --- | --- |
| *S.No* | *Entrez Gene* | *Gene Symbol* | *Gene Title* | *RefSeq Transcript ID* | *Fold change in HUVECs* | *Regulation in HUVECs* | *Fold change in BCBL1* | *Regulation in BCBL1* |
| 1. | 10537 | UBD | ubiquitin D | NM_001470 | 61.0 | up | 26.0 | up |
| 2. | 7412 | VCAM1 | vascular cell adhesion molecule 1 | NM_001078 | 48.3 | up | 5.0 | up |
| 3. | 3627 | CXCL10 | chemokine (C-X-C motif) ligand 10 | NM_001565 | 47.3 | up | 3.5 | up |
| 4. | 6352 | CCL5 | chemokine (C-C motif) ligand 5 | NM_002985 | 49.7 | up | 9.4 | up |
| 5. | 6376 | CX3CL1 | chemokine (C-X3-C motif) ligand 1 | NM_002996 | 27.4 | up | 3.4 | up |
| 6. | 7128 | TNFAIP3 | tumor necrosis factor, alpha-induced protein 3 | NM_006290 | 24.1 | up | 12.8 | up |
| 7. | 3383 | ICAM1 | intercellular adhesion molecule 1 | NM_000201 | 20.4 | up | 4.9 | up |
| 8. | 10148 | EBI3 | Epstein-Barr virus induced 3 | NM_005755 | 14.3 | up | 9.9 | up |
| 9. | 1520 | CTSS | cathepsin S | NM_004079 | 10.7 | up | 2.2 | up |
| 10. | 4792 | NFKBIA | nuclear factor of kappa light polypeptide gene enhancer in B-cells inhibitor | NM_020529 | 9.7 | up | 8.3 | up |
| 11. | 330 | BIRC3 | baculoviral IAP repeat-containing 3 | NM_001165 | 8.0 | up | 13.5 | up |
| 12. | 64332 | NFKBIZ | nuclear factor of kappa light polypeptide gene enhancer in B-cells inhibitor | NM_001005474 | 7.4 | up | 2.8 | up |
| 13. | 9235 | IL32 | interleukin 32 | NM_001012631 | 6.0 | up | 4.8 | up |
| 14. | 116496 | FAM129A | family with sequence similarity 129, member A | NM_052966 | 6.7 | up | 2.2 | up |
| 15. | 3437 | IFIT3 | interferon-induced protein with tetratricopeptide repeats 3 | NM_001031683 | 5.8 | up | 2.0 | up |
| 16. | 10318 | TNIP1 | TNFAIP3 interacting protein 1 | NM_006058 | 5.2 | up | 2.2 | up |
| 17. | 3134 | HLA-F | major histocompatibility complex, class I, F | NM_001098478 | 4.8 | up | 2.5 | up |
| 18. | 57007 | CXCR7 | chemokine (C-X-C motif) receptor 7 | NM_020311 | 4.6 | up | 2.0 | up |
| 19. | 27074 | LAMP3 | lysosomal-associated membrane protein 3 | NM_014398 | 4.2 | up | 3.9 | up |
| 20. | 259307 | IL4I1 | interleukin 4 induced 1 | NM_152899 | 4.0 | up | 6.5 | up |
| 21. | 285628 | LOC285628 | hypothetical protein LOC285628 | NA | 3.8 | up | 3.0 | up |
| 22. | 3726 | JUNB | jun B proto-oncogene | NM_002229 | 3.8 | up | 2.7 | up |
| 23. | 5366 | PMAIP1 | phorbol-12-myristate-13-acetate-induced protein 1 | NM_021127 | 3.6 | up | 2.1 | up |
| 24. | 64135 | IFIH1 | interferon induced with helicase C domain 1 | NM_022168 | 2.9 | up | 2.7 | up |
| 25. | 8870 | IER3 | immediate early response 3 | NM_003897 | 2.8 | up | 3.4 | up |
| 26. | 4050 | LTB | lymphotoxin beta (TNF superfamily, member 3) | NM_002341 | 2.7 | up | 12.9 | up |
| 27. | 4794 | NFKBIE | nuclear factor of kappa light polypeptide gene enhancer in B cells inhibitor | NM_004556 | 2.6 | up | 3.0 | up |
| 28. | 3604 | TNFRSF9 | tumor necrosis factor receptor superfamily, member 9 | NM_001561 | 2.5 | up | 2.7 | up |
| 29. | 22898 | DENND3 | DENN/MADD domain containing 3 | NM_014957 | 2.3 | up | 3.4 | up |
| 30. | 5971 | RELB | v-rel reticuloendotheliosis viral oncogene homolog B | NM_006509 | 2.3 | up | 4.9 | up |
| 31. | 25797 | QPCT | glutaminyl-peptide cyclotransferase | NM_012413 | 2.2 | up | 2.1 | up |
| 32. | 972 | CD74 | CD74 molecule, major histocompatibility complex, class II invariant chain | NM_001025158 | 2.1 | up | 7.0 | up |
| 33. | 3566 | IL4R | interleukin 4 receptor | NM_000418 | 2.1 | up | 2.1 | up |
| 34. | 1466 | CSRP2 | cysteine and glycine-rich protein 2 | NM_001321 | 2.0 | up | 2.6 | up |
| 35. | 6892 | TAPBP | TAP binding protein (tapasin) | NM_003190 | 2.0 | up | 4.1 | up |
| 36. | 4814 | NINJ1 | ninjurin 1 | NM_004148 | 2.0 | up | 2.8 | up |
| 37. | 9641 | IKBKE | inhibitor of kappa light polypeptide gene enhancer in B-cells | NM_014002 | 2.0 | up | 2.9 | up |
| 38. | 3487 | IGFBP4 | insulin-like growth factor binding protein 4 | NM_001552 | 2.4 | down | 3.0 | up |
| 39. | 1543 | CYP1A1 | cytochrome P450, family 1, subfamily A, polypeptide 1 | NM_000499 | 2.4 | down | 6.0 | up |
| 40. | 3855 | KRT7 | keratin 7 | NM_005556 | 2.1 | down | 2.4 | up |
| 41. | 7852 | CXCR4 | chemokine (C-X-C motif) receptor 4 | NM_001008540 | 2.1 | down | 3.0 | up |
| 42. | 5567 | PRKACB | protein kinase, cAMP-dependent, catalytic, beta | NM_002731 | 2.0 | down | 2.1 | down |
