# Supplementary material for: A Computational Profiling of Changes in Gene Expression and Transcription Factors Induced by vFLIP K13 in Primary Effusion Lymphoma
Source: PLoS One. 2012 May 18;7(5):e37498. doi: 10.1371/journal.pone.0037498 (PMC3356309; doi:10.1371/journal.pone.0037498)
Supplement: Table S4 — List of genes upregulated >20 fold in HUVECs dataset. (DOC) [file pone.0037498.s004.doc]

| *Table S4. List of genes upregulated > 20 fold in HUVECs dataset.* | | | | | | |
| --- | --- | --- | --- | --- | --- | --- |
| *S.No* | *Entrez Gene* | *Gene Symbol* | *Gene Title* | *RefSeq Transcript ID* | *Fold change* | *Regulation* |
| 1. | 6347 | CCL2 | chemokine (C-C motif) ligand 2 | NM_002982 | 73.06 | up |
| 2. | 10537 | UBD | ubiquitin D | NM_006398 | 71.02 | up |
| 3. | 6364 | CCL20 | chemokine (C-C motif) ligand 20 | NM_004591 | 63.87 | up |
| 4. | 7412 | VCAM1 | vascular cell adhesion molecule 1 | NM_001078 | 58.30 | up |
| 5. | 3627 | CXCL10 | chemokine (C-X-C motif) ligand 10 | NM_001565 | 53.32 | up |
| 6. | 6352 | CCL5 | chemokine (C-C motif) ligand 5 | NM_002985 | 49.73 | up |
| 7. | 6401 | SELE | selectin E | NM_000450 | 38.56 | up |
| 8. | 84419 | C15orf48 | chromosome 15 open reading frame 48 | NM_197955 | 35.27 | up |
| 9. | 2921 | CXCL3 | chemokine (C-X-C motif) ligand 3 | NM_002090 | 34.80 | up |
| 10. | 6372 | CXCL6 | chemokine (C-X-C motif) ligand 6 | NM_002993 | 33.39 | up |
| 11. | 6376 | CX3CL1 | chemokine (C-X3-C motif) ligand 1 | NM_002996 | 30.48 | up |
| 12. | 7130 | TNFAIP6 | tumor necrosis factor, alpha-induced protein 6 | NM_007115 | 24.31 | up |
| 13. | 7128 | TNFAIP3 | tumor necrosis factor, alpha-induced protein 3 | NM_006290 | 24.15 | up |
| 14. | 2919 | CXCL1 | chemokine (C-X-C motif) ligand 1 | NM_001511 | 23.46 | up |
