# Supplementary material for: A Computational Profiling of Changes in Gene Expression and Transcription Factors Induced by vFLIP K13 in Primary Effusion Lymphoma
Source: PLoS One. 2012 May 18;7(5):e37498. doi: 10.1371/journal.pone.0037498 (PMC3356309; doi:10.1371/journal.pone.0037498)
Supplement: Table S5 — Summary of representative genes corresponding to pathways identified by GSEA analysis and validated in qRT-PCR. (DOCX) [file pone.0037498.s005.docx]

| *Table S5. Summary of representative genes corresponding to pathways identified by GSEA analysis and validated in qRT-PCR.* | | | |
| --- | --- | --- | --- |
| *Pathway* | *Gene* | *Enetrz gene ID* | *Rank in GSEA* |
| Cytokine pathway | CCL5 (chemokine (C-C motif) ligand 5) | 6352 | 20591 |
|  | CXCL10 (chemokine (C-X-C motif) ligand 10) | 3627 | 20582 |
|  | Interleukin 9 (IL9) | 3578 | 20580 |
|  | Interleukin 6 (IL6) | 3569 | 20395 |
|  | Interferon gamma (IFNG) | 3458 | 19998 |
|  | Interleukin 15 (IL15) | 3600 | 19284 |
|  | LTB (lymphotoxin beta receptor (TNFR superfamily, member 3) | 4055 | 20573 |
| NF-κB pathway | NFKBIA (p65, nuclear factor of kappa light polypeptide gene enhancer in B-cells inhibitor, alpha) | 4792 | 20573 |
|  | NFKB1(p105, nuclear factor of kappa light polypeptide gene enhancer in B-cells 1 ) | 4790 | 20166 |
|  | LMNB2 (lamin B2) | 84823 | 20017 |
|  | TRADD (TNFRSF1A-associated via death domain) | 8717 | 19867 |
|  | TNFRSF1B (tumor necrosis factor receptor superfamily, member 1B) | 7133 | 19287 |
| Cell death pathway | CCL5 (chemokine (C-C motif) ligand 5) | 6352 | 20591 |
|  | BIRC3 (baculoviral IAP repeat-containing 3) | 330 | 20583 |
|  | NFKBIA (p65, nuclear factor of kappa light polypeptide gene enhancer in B-cells inhibitor, alpha) | 4792 | 20573 |
|  | NFKB1(p105, nuclear factor of kappa light polypeptide gene enhancer in B-cells 1 ) | 4790 | 20166 |
|  | TRADD (TNFRSF1A-associated via death domain) | 8717 | 19867 |
|  | TNFRSF25 (tumor necrosis factor receptor superfamily, member 25) | 8718 | 19453 |
|  | GAS2 (growth arrest-specific 2) | 2620 | 19272 |
|  | BID (BH3 interacting domain death agonist) | 637 | 19052 |
| Cell Adhesion | VCAM1 (vascular cell adhesion molecule 1) | 7412 | 20595 |
|  | HLA-DQA1 (major histocompatibility complex, class II, DQ alpha 1) | 3117 | 20567 |
|  | HLA-DMB (major histocompatibility complex, class II, DM beta) | 3109 | 20549 |
|  | HLA-DQB1 (major histocompatibility complex, class II, DQ beta 1) | 3119 | 20508 |
|  | SELE (selectin E (endothelial adhesion molecule 1)) | 6401 | 20464 |
|  | ALCAM (activated leukocyte cell adhesion molecule) | 214 | 19756 |
| Antigen Processing | HLA-DQA1 (major histocompatibility complex, class II, DQ alpha 1) | 3117 | 20567 |
|  | CIITA (class II, major histocompatibility complex, transactivator) | 4261 | 20557 |
|  | CD74 (CD74 molecule, major histocompatibility complex, class II invariant chain) | 972 | 20553 |
|  | HLA-DMB (major histocompatibility complex, class II, DM beta) | 3109 | 20549 |
|  | HLA-DQB1 (major histocompatibility complex, class II, DQ beta 1) | 3119 | 20508 |
|  | CTSS ( cathepsin S) | 1520 | 20215 |
